# Electrospun Polymeric Nanofibers Incorporating Brazilian Red Propolis Extract for Wound Dressing Applications

**DOI:** 10.3390/pharmaceutics18070888

**Published:** 2026-07-20

**Authors:** Maria Sirlene Morais, Paulo Augusto Marques Chagas, Gustavo Cardoso da Mata, Gabriela Rodrigues Silva, Elaine Cristina Pereira De Martinis, Guilherme Henrique Alves Pinto, Gabriela Fávero Galvão, Monica Lopes Aguiar, Wanderley Pereira Oliveira

**Affiliations:** 1 School of Pharmaceutical Sciences of Ribeirão Preto, University of São Paulo, Ribeirão Preto 14040-903, SP, Brazil; paulochagas@ufscar.br (P.A.M.C.); silva_gabriela@usp.br (G.R.S.); edemarti@usp.br (E.C.P.D.M.); guilhermealvespinto@usp.br (G.H.A.P.); gabrielafgalvao@usp.br (G.F.G.); 2 Department of Chemical Engineering, Federal University of São Carlos, São Carlos 13565-905, SP, Brazil; gugs_cardoso@ufsj.edu.br (G.C.d.M.); mlaguiar@ufscar.br (M.L.A.)

**Keywords:** Brazilian red propolis extract, electrospinning, nanofibers, wound dressing, antimicrobial activity, cytocompatibility

## Abstract

**Background/Objectives**: Chronic wounds remain difficult to manage because persistent inflammation, microbial colonization, and excess exudate require dressings that combine structural integrity, bioactivity, antimicrobial performance, and cytocompatibility. This study aimed to develop electrospun nanofibrous mats based on gelatin, poly(vinyl alcohol) (PVA), and poly(ε-caprolactone) (PCL), with and without Brazilian red propolis extract (BRPE), and to evaluate how extract incorporation affects solution properties, fiber morphology, fluid interaction, antimicrobial activity, and cytocompatibility. **Methods**: BRPE was characterized in terms of solid content, total phenolic content, antioxidant activity, and HPLC-DAD marker profile. Polymeric solutions were evaluated for electrical conductivity and rheological behavior and then processed by electrospinning under fixed conditions. The resulting mats were characterized by scanning electron microscopy, surface porosity, FTIR, and HPLC-DAD. Their performance was further assessed by swelling-associated degradation in simulated wound fluids, agar diffusion antimicrobial assays, and MTT cytocompatibility assays using HaCaT cells. **Results**: BRPE showed a solid content of 3.88%, a total phenolic content of 8.79 ± 0.21 mg pyrogallol equivalents g^−1^ extract, and an antioxidant activity of 75.32 ± 9.80 mg Trolox equivalents g^−1^ extract. HPLC-DAD confirmed preservation of the BRPE chromatographic fingerprint after electrospinning, with high retention of marker peaks associated with liquiritigenin and a formononetin-related signal. Solution conductivity varied with polymer composition and BRPE incorporation; for example, the PVA:gelatin:PCL formulation A4/A4.1 at 70:20:10 decreased from 919.6 to 539.6 µS cm^−1^ after BRPE loading. Electrospinning produced continuous, defect-free fibers with mean diameters ranging from 94 to 224 nm and surface porosity between 9.8 and 10.6%. Most hydrophilic systems showed rapid fluid interaction but limited wet-state structural stability; among the quantified formulations, A5 showed the lowest mass loss, indicating better structural preservation under simulated wound conditions. BRPE-loaded mats showed microorganism-dependent antimicrobial activity, with the strongest inhibition against *Staphylococcus epidermidis* and *Klebsiella pneumoniae* and no activity against *Pseudomonas aeruginosa*. Free BRPE showed marked cytotoxicity, whereas selected electrospun formulations, especially A1.1 and A3.1, improved HaCaT cell viability. **Conclusions**: Electrospinning was an effective strategy for incorporating BRPE into polymeric nanofibers and modulating the physicochemical and biological performance of the resulting mats. These findings support the potential of these materials as multifunctional wound-dressing platforms, although further optimization is needed to improve wet-state structural stability, mechanical performance, and bioactive release.

## 1. Introduction

Chronic wounds represent a major global healthcare challenge because they fail to progress through the normal and orderly phases of healing within the expected timeframe. They are commonly associated with underlying conditions such as diabetes, vascular diseases, and persistent inflammatory disorders, all of which impair tissue regeneration and reduce quality of life [1,2]. In addition to their clinical consequences, chronic wounds impose a substantial economic burden on healthcare systems, highlighting the need for more effective and multifunctional therapeutic strategies [1].

A major factor contributing to delayed wound healing is the persistence of a hostile wound microenvironment, characterized by altered pH, reduced oxygen and nutrient availability, excess exudate, and sustained inflammation [3]. Under these conditions, microbial biofilms can develop and stabilize on the wound surface. These structured microbial communities, embedded in a self-produced extracellular polymeric matrix, are highly resistant to host defense mechanisms and conventional antimicrobial therapies, thereby prolonging inflammation and further compromising tissue repair [4,5]. Therefore, advanced wound dressings should not only protect the lesion mechanically, but also help control moisture, prevent microbial contamination, support gas exchange, and create conditions favorable for tissue regeneration [6,7].

In recent years, nanotechnology-based platforms, including nanofibers, hydrogels, and nanoparticles, have emerged as promising alternatives to conventional wound dressings [3,6]. Among them, electrospun nanofibers have attracted particular interest because they combine structural versatility with relatively simple processing [8,9]. By applying a high-voltage electric field to a polymer solution, electrospinning produces continuous fibers with diameters in the micro- to nanoscale range and enables fine control over morphology, composition, and surface characteristics. Electrospun fiber mats typically exhibit a high surface-area-to-volume ratio, interconnected porosity, and structural similarity to the native extracellular matrix, features that are advantageous for cell adhesion, proliferation, and nutrient transport in wound care applications [10].

The performance of electrospun wound dressings depends strongly on the choice and combination of polymers. In this context, blends of natural and synthetic polymers are especially attractive because they allow complementary properties to be integrated within a single material [10,11]. Gelatin, derived from collagen, contributes biocompatibility and biological recognition; poly(vinyl alcohol) (PVA) offers hydrophilicity and good electrospinnability; and poly(ε-caprolactone) (PCL) provides mechanical strength and a tunable degradation profile [12,13,14,15]. Together, these polymers can generate multifunctional fibrous matrices with physicochemical and biological properties that are relevant for wound dressing applications.

Natural products have also received growing attention as sources of multifunctional bioactive agents for wound management. Brazilian red propolis is particularly interesting because of its rich and complex phytochemical composition, including flavonoids and isoflavonoids such as liquiritigenin and formononetin, which have been associated with antioxidant, antimicrobial, and anti-inflammatory activities [16,17,18]. These attributes make red propolis a promising candidate for incorporation into bioactive wound dressings. However, the use of complex natural extracts in electrospun systems remains challenging, since their incorporation may alter solution behavior, fiber formation, physicochemical stability, and biological performance.

Previous studies have demonstrated the feasibility of incorporating propolis into electrospun matrices for wound-related applications, including PVA nanoscaffolds associated with propolis nanoparticles [19], PCL-based propolis mats with different morphologies [20], PVA- or PCL-based propolis-loaded nanofiber scaffolds [21], PVA/chitosan/propolis/nystatin wound dressing mats [12], and zein-based fibers containing ethanolic propolis extract [22]. In addition, propolis-containing electrospun polymeric composites have been reported as antioxidant bioactive platforms, further supporting the compatibility of propolis extracts with electrospinning-based systems [23]. These studies collectively show that propolis can improve antioxidant, antimicrobial, and wound-relevant biological properties of electrospun materials. However, most available reports have focused on green propolis or non-Brazilian propolis sources, propolis nanoparticles, isolated polymer systems, or binary matrices. In contrast, the incorporation of native Brazilian red propolis extract (BRPE), in its chemically complex form, into blended gelatin/PVA/PCL electrospun matrices remains insufficiently explored. In particular, limited information is available on how native BRPE affects solution conductivity, rheological behavior, electrospinnability, fiber morphology, fluid interaction, antimicrobial response, and cytocompatibility. Addressing this gap is important because the rational design of multifunctional wound-dressing platforms depends not only on the biological activity of the extract itself, but also on how its incorporation influences the structure and performance of the fibrous carrier.

Therefore, the aim of this study was to develop electrospun nanofibrous mats based on gelatin, poly(vinyl alcohol), and poly(ε-caprolactone), with and without BRPE, and to evaluate their potential as multifunctional wound dressing platforms. The study investigated the effect of extract incorporation on solution properties, fiber morphology, molecular interactions, fluid uptake/degradation behavior, antimicrobial activity, and cytocompatibility. Overall, the results show that electrospinning enabled the incorporation of BRPE into continuous nanofibers and modulated the physicochemical and biological performance of the resulting mats, although further optimization is still required to improve their structural stability under simulated wound conditions.

## 2. Materials and Methods

### 2.1. Materials and Reagents

Raw Brazilian red propolis was supplied by Rubee Apis Sociedade Agrícola Klieber LTDA (Barra de Santo Antônio, AL, Brazil), and registered in SisGen under code AE31FC2. Ethanol (96%, *v*/*v*), glacial acetic acid (Perfyl Tech, São Bernardo do Campo, SP, Brazil; lot 8144, ref. RP 001051), and MilliQ purified water were used as solvents. The polymers employed for membrane production were bovine gelatin type B (Sigma-Aldrich, St. Louis, MO, USA; G931), poly(vinyl alcohol) (PVA; Neon, Suzano, SP, Brazil; Ref. 02565, molecular weight 104,500 g mol^−1^, medium viscosity), and poly(ε-caprolactone) (PCL; Sigma-Aldrich, St. Louis, MO, USA; 440744). Simulated wound fluid A (SWF A) and Solution A were prepared using sodium chloride (NaCl, P.A., Labsynth, Diadema, SP, Brazil), calcium chloride (CaCl_2_, Dinâmica, Indaiatuba, SP, Brazil), potassium chloride (KCl, Labsynth, Diadema, SP, Brazil), magnesium chloride (MgCl_2_, Labsynth, Diadema, SP, Brazil), bovine serum albumin (BSA, lyophilized powder, ≥96%, Sigma-Aldrich, St. Louis, MO, USA), potassium phosphate (KH2PO4, Labsynth, Diadema, SP, Brazil), and sodium bicarbonate (NaHCO_3_, Cetus Indústria e Comércio de Produtos Químicos Ltda, São Paulo, SP, Brazil) according to the composition described in Table 1.

Solution A was prepared according to EN 13726. Briefly, 8.298 g of NaCl and 0.368 g of CaCl_2_ were dissolved in approximately 800 mL of purified water under magnetic stirring. The solution was then transferred to a 1 L volumetric flask, and the volume was adjusted with purified water. SWF A was prepared according to Svensby et al. [24] by dissolving the components listed in Table 1 in purified water under magnetic stirring until complete solubilization. Both media were then filtered, and their pH and electrical conductivity were measured at room temperature using a pH meter (Model 827, Metrohm AG, Herisau, Switzerland) and a Metrohm 912 bench-top conductometer (Metrohm AG, Herisau, Switzerland), respectively.

### 2.2. Preparation of Brazilian Red Propolis Extract

Brazilian red propolis extract (BRPE) was prepared by dynamic maceration using ethanol (96%, *v*/*v*) as the extraction solvent. Although hydroethanolic mixtures containing 70–80% ethanol are commonly used for propolis extraction, the use of 96% (*v*/*v*) ethanol was selected in the present study because Brazilian red propolis contains flavonoids and isoflavonoids of relatively low to intermediate polarity, such as liquiritigenin, formononetin, vestitol, neovestitol, medicarpin, and related phenolic markers [18,25,26]. Therefore, a high-ethanol solvent system was considered suitable to favor extraction of these constituents while reducing the extraction of highly hydrophilic impurities. In addition, the lower water content of 96% (*v*/*v*) ethanol minimized water introduction into the electrospinning polymer solutions, improving compatibility with the selected acetic-acid-based solvent system. Briefly, raw propolis (25 g) was extracted with 625 mL of ethanol, corresponding to a 1:25 (*w*/*v*) raw propolis-to-solvent ratio, under dynamic maceration at 62 °C for 2 h in a jacketed stirred vessel coupled to a thermostatic bath. After extraction, the mixture was vacuum-filtered through filter paper to remove insoluble, non-extractable residues, mainly beeswax, and the filtrate was stored at 8 °C until use. The filtrate was subsequently used for physicochemical characterization, including determination of solid content, total phenolic content, and antioxidant activity, as well as for preparation of the electrospinning formulations. The protocols used in these physicochemical characterizations are presented as follows.

#### 2.2.1. Solids Content

The solid content of BRPE was determined in triplicate by drying 2.0 g aliquots to constant mass at 105 °C using a moisture analyzer (MA35M, Sartorius AG, Göttingen, Germany), with constant mass defined as a mass variation lower than 1 mg/min. The results were expressed as a percentage of solids on a mass basis and calculated according to Equation (1):

*Cs* = *m*/*P* (1)

where *Cs* is the solid content (% *w*/*w*), m  is the mass of dried residue (g), and P  is the initial mass of the extract sample (g).

#### 2.2.2. Determination of Total Phenolic Content

The total phenolic content (TPC) of BRPE was determined by UV–Vis spectrophotometry using an HP 8453 spectrophotometer operated with HP ChemStation software Rev. A.10.02 (Agilent Life Sciences and Chemical Analysis, Santa Clara, CA, USA). Quantification was based on the reduction of the phosphomolybdic–phosphotungstic acid complex of the Folin–Denis reagent by phenolic compounds under alkaline conditions, according to Cortés-Rojas et al. [27]. The reaction medium consisted of 0.5 mL of diluted extract, 0.5 mL of Folin-Denis reagent, and 4.0 mL of a 20% sodium carbonate solution, and the absorbance of the sample at 750 nm was analyzed after 2 min. A pyrogallol calibration curve was prepared, and the results were expressed as milligrams of pyrogallol equivalents per gram of extract on a dry basis (mg PGE/g extract, d.b.). All determinations were performed in triplicate.

#### 2.2.3. Determination of Antioxidant Activity by the DPPH Assay

The antioxidant activity of BRPE was evaluated by the DPPH radical scavenging assay according to Georgetti et al. [28]. The results were expressed as Trolox equivalents based on a calibration curve. The reaction medium consisted of 1.0 mL of acetate buffer (0.1 M, pH 5.5), 1.0 mL of ethanol, and 10 µL of the extract solution diluted in ethanol. The reduction of the DPPH radical was monitored by measuring the absorbance at 517 nm after 20 min of reaction. All measurements were performed in triplicate.

#### 2.2.4. HPLC-DAD Fingerprinting and Quantification of Marker Compounds

High-performance liquid chromatography with diode-array detection (HPLC-DAD) was used to obtain the chromatographic fingerprint of Brazilian red propolis extract (BRPE) and to monitor two characteristic phenolic marker peaks associated with liquiritigenin (LQ) and formononetin (FN). These compounds were selected because they are frequently reported among the characteristic flavonoid and isoflavonoid constituents of Brazilian red propolis, together with other phenolic markers such as calycosin, isoliquiritigenin, vestitol, neovestitol, medicarpin, biochanin A, and 7-O-methylvestitol [17,18]. The chromatographic method was based on the procedure described by Aldana-Mejía et al. [17], with modifications in chromatographic conditions, detection wavelength, and sample preparation.

Chromatographic analyses were performed using a Shimadzu Prominence LC-20A system coupled to an LC-6A dual pump (Shimadzu Corporation, Kyoto, Japan). Separation was achieved on a C18 column (Shimadzu Shim-Pack CLC(M), 4.6 mm × 25 cm, 5 µm, 100 Å) maintained at 45 °C. The mobile phase consisted of purified water containing 0.1% formic acid (A) and acetonitrile (B), using the following gradient program: 0–10 min, 20% B; 10–30 min, linear increase to 50% B; 31–40 min, linear increase to 80% B; 40–41 min, 80% B; 41–51 min, linear increase to 100% B; 51–61 min, 100% B; 61–62 min, linear decrease to 20% B; and 62–70 min, 20% B for column re-equilibration. Chromatograms were recorded at 281 nm, which provided suitable resolution for the selected marker peaks.

For BRPE analysis, 66 µL of native extract was diluted with methanol to a final volume of 2.0 mL, corresponding to a final concentration of 1 mg/mL based on dry extract solids. The solution was filtered through a 0.45 µm PVDF membrane filter and transferred to chromatographic vials. The injection volume was 20 µL, and BRPE analyses were performed in duplicate. For BRPE-loaded nanofiber analysis, electrospun mats were cut into fragments, and 15 mg of each sample was transferred to 3 mL of methanol. The dispersions were kept under magnetic stirring for approximately 4 h to extract the incorporated compounds. The resulting solutions were filtered through a 0.45 µm PVDF membrane filter, transferred to amber vials, and analyzed under the same chromatographic conditions used for BRPE. The injection volume was 20 µL, and nanofiber analyses were performed in triplicate.

Peak assignment was performed by comparison with authentic standards analyzed under identical chromatographic conditions, primarily based on retention time and, when possible, supported by DAD UV spectral comparison, following the general selectivity criteria adopted for phenolic profiling by HPLC-DAD [17,29]. Under the present analytical conditions, the peak assigned to LQ showed agreement with the authentic standard in both retention time and UV spectrum. For the peak assigned to FN, retention time was consistent with that of the authentic standard, and peak-purity analysis indicated a highly homogeneous signal. This peak was therefore used as the FN-associated marker peak for comparative and quantitative monitoring of BRPE and BRPE-loaded nanofibers.

Quantification was performed using analytical calibration curves in the range of 1–100 µg/mL. The regression equations were y = 40,032x − 18,169 for LQ and y = 32,648x − 35,050 for FN, with coefficients of determination (R^2^) of 0.9981 and 0.9992, respectively. Theoretical marker contents were calculated from BRPE concentrations and nominal extract loading in the formulations (10% *w*/*w*, relative to total polymer mass). Marker retention efficiency (RE, %) was calculated according to Equation (2):

RE (%) = (experimental marker content/theoretical marker content) × 100(2)

### 2.3. Preparation of Electrospinning Solutions

Polymeric stock solutions were prepared separately. PVA was dissolved in 70% (*v*/*v*) aqueous acetic acid under magnetic stirring at 90 °C for 4 h to obtain a 12% (*w*/*v*) solution. Gelatin was dissolved in 90% (*v*/*v*) acetic acid under magnetic stirring for 2 h to obtain a 12% (*w*/*v*) solution. PCL was dissolved in 90% (*v*/*v*) acetic acid under stirring at room temperature for 4 h to obtain a 20% (*w*/*v*) solution. The polymeric solutions were then mixed according to the volume ratios described in Table 2. The amount of BRPE incorporated into the polymeric solutions was calculated based on its solid content (3.88%), aiming at a final concentration of 10% (*w*/*w*) relative to the total polymer mass. All solutions were cooled to room temperature before electrospinning. The resulting formulations were subsequently characterized for rheological behavior and electrical conductivity, since both properties influence fiber formation during electrospinning.

#### 2.3.1. Electrical Conductivity Measurements

The electrical conductivity of the electrospinning solutions was measured at room temperature using a Metrohm 912 bench-top conductometer (Metrohm AG, Herisau, Switzerland). Formulations prepared without Brazilian red propolis extract were considered blank formulations. All measurements were performed in triplicate, and the results were expressed as means ± standard deviations.

#### 2.3.2. Rheological Analysis

Rheological measurements of the formulations were performed using a Brookfield LV-DVIII coaxial-cylinder rheometer (Brookfield Engineering Laboratories Inc., Middleboro, MA, USA) equipped with a small sample adapter and SC4-18 or SC4-25 spindles, depending on sample viscosity. Each sample was transferred to the small sample adapter, and the corresponding spindle was coupled to the instrument. Shear rate was then increased according to the programmed method, while Brookfield Rheocalc 3.2 software controlled the equipment and recorded the corresponding shear stress data. The flow behavior of the formulations was described using the Ostwald–de Waele power-law model (Equation (3)), where τ is the shear stress (Pa), K is the consistency index (Pa·s^n^), γ is the shear rate (s^−1^), and *n* is the flow behavior index [12]:

(3) $$\tau =K\cdot \gamma^{n}$$

### 2.4. Electrospinning Apparatus and Processing Conditions

Electrospinning was performed using a setup consisting of a high-voltage power supply (0 to 50 kV, HIPOT CC, Model EH6005C; Eletroteste, Santa Rita do Sapucaí, MG, Brazil), a syringe pump (Pump 11 Elite, Infusion/Withdrawal Programmable Single Syringe; Harvard Apparatus, Holliston, MA, USA), and a grounded stainless-steel cylindrical collector with a diameter of 100 mm and length of 200 mm, covered with aluminum foil. The polymeric formulations were loaded into 5 mL syringes fitted with metallic needles with an internal diameter of 0.7 mm. The needle was connected to the positive terminal of the high-voltage source, while the collector was connected to the ground/negative terminal.

The electrospinning parameters were fixed at an applied voltage of 22 kV, a tip-to-collector distance of 10 cm, and a feed flow rate of 0.5 mL/h. The formulations were electrospun for 3 h under these conditions. The resulting nanofiber mats were collected on aluminum foil, removed from the collector, and weighed. The selection of these operating conditions was based on preliminary tests aimed at obtaining continuous electrospun nanofibers with minimal defects.

### 2.5. Physicochemical Characterization of Electrospun Nanofiber Mats

#### 2.5.1. Morphology by Scanning Electron Microscopy

The morphology of the electrospun nanofiber mats was evaluated by scanning electron microscopy (SEM) using a FEI Magellan 400 L field-emission gun microscope (FEI Company, Hillsboro, OR, USA). Prior to analysis, the samples were fixed on aluminum stubs using double-sided carbon tape and sputter- with a thin layer of gold using a Balzers Union SCD 004/CED 020 sputter-coating system (Balzers, Liechtenstein) to improve electrical conductivity. Representative micrographs were obtained at appropriate magnifications for qualitative assessment of fiber continuity, uniformity, and the presence of defects such as beads or fused regions.

#### 2.5.2. Fiber Diameter and Surface Porosity Estimated from SEM Images

Fiber diameter and surface porosity were determined from SEM images using ImageJ software version 1.53k (National Institutes of Health, USA). For fiber diameter analysis, SEM images acquired at 80,000× magnification were calibrated using the scale bar provided in each micrograph through the Set Scale function. For each sample, 100 fibers were randomly selected and measured across different regions of representative micrographs to minimize local bias and ensure adequate sampling of the fibrous network. The resulting data were exported and analyzed using Origin 2025 software (OriginLab Corporation, Northampton, MA, USA) to obtain the mean fiber diameter and standard deviation.

Surface porosity was estimated by digital image analysis of SEM micrographs acquired at 10,000× magnification using ImageJ. The images were first converted to 8-bit grayscale and calibrated using the scale bar provided in each micrograph. A consistent thresholding procedure was applied to distinguish fibers from void regions. Surface porosity was then calculated according to Equation (4):

(4) $$\mathrm{Surface}\;\mathrm{porosity}\;(\%)=\frac{\mathrm{total}\;\mathrm{pore}\;\mathrm{area}}{\mathrm{total}\;\mathrm{analyzed}\;\mathrm{image}\;\mathrm{area}}\times 100$$

Because this parameter is derived from two-dimensional SEM images, it should be interpreted as apparent surface porosity estimated from SEM micrographs, rather than total porosity of the electrospun mat.

#### 2.5.3. Fourier Transform Infrared Spectroscopy (FTIR)

The chemical structure of the electrospun nanofiber mats was analyzed by Fourier transform infrared spectroscopy (FTIR) using a Bruker Tensor 27 spectrometer (Bruker Optik GmbH, Ettlingen, Germany). Spectra were recorded over the range of 4000 to 400 cm^−1^. The spectra were used to identify the main functional groups of the polymeric matrix and to evaluate possible spectral changes associated with incorporation of Brazilian red propolis extract.

#### 2.5.4. Swelling and Coupled Swelling–Degradation Assessment

The fluid interaction behavior of the electrospun mats was evaluated in Solution A and simulated wound fluid A (SWF A) at 37 °C. Samples were cut into standardized dimensions (1 × 2 cm^2^) and weighed to determine their initial dry mass ($W_{d}$). Each sample was then exposed to a defined volume (1 mL) of test medium sufficient to ensure sink conditions and rapid hydration.

At predetermined time points (10 and 30 min), excess surface liquid was carefully removed with absorbent paper without applying pressure, and the swollen mass ($W_{w}$) was recorded. Water uptake was calculated according to Equation (5):

(5) $$\mathrm{Water}\;\mathrm{uptake}\;(\%)=\frac{W_{w}-W_{d}}{W_{d}}\times 100$$

where $W_{w}\;$ is the swollen mass and $W_{d\;}$ is the initial dry mass.

Because the formulations were highly hydrophilic, swelling and the onset of structural degradation were expected to occur simultaneously. Therefore, in addition to water uptake, the samples were monitored for mass loss after immersion. After the final time point, the samples were dried in an oven at 37 °C for 24 h and weighed to determine the final dry mass ($W_{df}$). Mass loss was calculated according to Equation (6):

(6) $$\mathrm{Mass}\;\mathrm{loss}\;(\%)=\frac{W_{d}-W_{df}}{W_{d}}\times 100$$

All experiments were performed in triplicate, and the results were expressed as means ± standard deviations.

### 2.6. Biological Evaluation of the Electrospun Mat

#### 2.6.1. Antimicrobial Activity

The antimicrobial activity of electrospun fibers containing Brazilian red propolis extract was evaluated by agar diffusion assay against *Staphylococcus epidermidis* ATCC 14990, *Escherichia coli* CDC O2A.2B, *Pseudomonas aeruginosa* ATCC 14502, *Staphylococcus aureus* ATCC 29213, *Enterococcus faecalis* 62, *Klebsiella pneumoniae* ATCC 10031, and *Candida albicans* ATCC 18804. Bacterial inocula were adjusted to the turbidity of a 0.5 McFarland standard, corresponding to approximately 1 × 10^8^ CFU/mL, and were spread uniformly onto Mueller–Hinton agar plates to obtain confluent growth. For *C. albicans*, Mueller–Hinton agar supplemented with 2% glucose was used. Fiber specimens were cut into circular sections (0.06 cm^2^), placed directly on the inoculated agar surface, and incubated in an inverted position at 35–37 °C for 24 h. A negative control consisting of fibers without extract was included. The diameters of the inhibition zones around the fiber specimens (ZOI) were measured and classified as total inhibition, defined as a clear zone without microbial growth, or partial inhibition, defined as a diffuse zone with reduced microbial growth. Inhibition-zone diameters were determined from digital plate images using the known 90 mm Petri dish diameter as an internal calibration reference. For irregular inhibition zones or samples showing material spreading, only the clearly defined circular portion of the inhibition zone was measured. The resulting diameters were interpreted as semi-quantitative estimates of antimicrobial activity. All assays were set up in triplicate; conditions with fewer than three valid measurements were reported as not determined (ND) and excluded from the quantitative analysis.

#### 2.6.2. Cytocompatibility by the MTT Assay

Cytocompatibility was evaluated by the MTT assay using immortalized human keratinocytes HaCaT (BCRJ-0341) originally purchased from the Rio de Janeiro Cell Bank (Banco de Células do Rio de Janeiro, BCRJ, Rio de Janeiro, RJ, Brazil) and maintained at the Cell Culture Laboratory of the School of Pharmaceutical Sciences of Ribeirão Preto, University of São Paulo (FCFRP-USP), Ribeirão Preto, SP, Brazil. Cells were cultured in Dulbecco’s Modified Eagle Medium (DMEM) supplemented with 10% (*v*/*v*) heat-inactivated fetal bovine serum and a 1% (*v*/*v*) antibiotic–antimycotic solution containing 10,000 IU/mL penicillin, 10 mg/mL streptomycin, and 25 µg/mL amphotericin B under a humidified atmosphere containing 5% CO_2_ at 37 °C.

For the assay, cells were seeded in 96-well plates at a density of 1 × 10^4^ cells/well and incubated for 24 h. The culture medium was then removed, and blank and BRPE-loaded electrospun mats, each with a circular area of 0.03 cm^2^, were placed in direct contact with the cells for an additional 24 h. Untreated cells were used as the cell viability control, 10% (*v*/*v*) DMSO was used as the positive control for cell death, and free BRPE was evaluated as the non-incorporated extract control.

After treatment, the wells were washed twice with phosphate-buffered saline (PBS), and MTT solution at 0.5 mg/mL was added. Following incubation for 4 h at 37 °C, the resulting formazan crystals were dissolved in 100% dimethyl sulfoxide (DMSO), and absorbance was measured at 540 nm using a Biotek Synergy HT microplate reader. Cell viability was expressed as a percentage relative to untreated cells. The assay was performed as one independent experiment with three technical replicates.

### 2.7. Statistical Analysis

Experimental results were expressed as mean ± standard deviation. Statistical analyses were performed using R software. The statistical tests applied for each dataset were selected according to the experimental design, data distribution, and type of comparison performed. Differences were considered statistically significant at *p* < 0.05. Unless otherwise stated, experiments were performed in triplicate (*n* = 3).

## 3. Results and Discussion

### 3.1. Characterization of Brazilian Red Propolis Extract

The Brazilian red propolis extract was characterized in terms of solid content, extraction efficiency, total phenolic content, and antioxidant activity to confirm its suitability as a bioactive input for the electrospinning formulations. The chemical composition of red propolis may vary according to the botanical source, geographic origin, seasonality, and extraction conditions, which directly influence its physicochemical and biological properties [18]. Despite this variability, studies have shown that Brazilian red propolis contains characteristic phenolic compounds that contribute substantially to its antioxidant and other biological properties, reinforcing the importance of phenolic profiling for quality control and standardization [16,17,18]. According to the Brazilian Technical Regulation for Identity and Quality of Propolis, established by the Ministry of Agriculture, Livestock, and Food Supply, compositional evaluation is important to ensure product quality and standardization [30]. In the present study, the native ethanolic extract of red propolis presented a solid content of 3.88%, indicating the concentration of extracted solids in the liquid extract obtained under the selected conditions. The extract also exhibited a total phenolic content of 8.79 ± 0.21 mg pyrogallol equivalents g^−1^ extract (dry basis) and an antioxidant activity of 75.32 ± 9.80 mg Trolox equivalents g^−1^ extract. Taken together, these results indicate that the extract used in this study contained relevant phenolic constituents and antioxidant capacity, supporting its use as the bioactive component of the electrospinning formulations.

### 3.2. Physicochemical Properties of Electrospinning Solutions

The physicochemical properties of electrospinning solutions are decisive for process stability and fiber formation, since they directly affect jet generation, stretching, and solidification under the applied electric field. Among these parameters, electrical conductivity and rheological behavior are particularly relevant because they govern charge transport and resistance to flow, respectively. In the present study, these properties were evaluated to understand how polymer composition and the incorporation of BRPE influenced the formulations’ processing behavior. This interpretation is linked to the electrospinning literature, which recognizes solution conductivity, viscosity, polymer concentration, and solvent characteristics as key variables controlling spinnability and the morphology of the resulting fibers [31]. Overall, the results demonstrate that both the solvent system and the presence of BRPE significantly modulated solution behavior, thereby affecting the electrospinning window of the developed systems.

#### 3.2.1. Electrical Conductivity

The electrical conductivity values of the electrospinning formulations are presented in Table 3, which replaces the original graphical presentation to provide a clearer comparison between each blank formulation and its corresponding BRPE-loaded system. Marked differences were observed among the samples, indicating that the polymer composition, solvent system, and BRPE incorporation affected the availability and mobility of ionic species in the solution. Among the blank formulations, A1 and A2 showed the highest conductivity values, followed by A3, A5, and A4. After BRPE incorporation, conductivity decreased in all corresponding loaded formulations, with reductions ranging from 36.0% to 55.8%. The largest decreases were observed for A1/A1.1 and A2/A2.1, followed by A5/A5.1 and A4/A4.1. For example, the conductivity of the PVA:gelatin:PCL formulation A4 decreased from 919.6 to 539.6 µS cm^−1^ after BRPE loading.

For comparison, the conductivity values of the individual reference solutions were 926.7 µS cm^−1^ for PVA/W, 810.8 µS cm^−1^ for gelatin/AA, 725.6 µS cm^−1^ for PVA/AA, 50.4 µS cm^−1^ for BRPE, and 10.8 µS cm^−1^ for PCL/AA. The relatively high conductivity of the PVA/gelatin blends is consistent with the contribution of the more conductive reference solutions, particularly PVA/W, gelatin/AA, and PVA/AA, whereas BRPE and PCL/AA exhibited substantially lower conductivities. Chi et al. [14] reported that gelatin/PVA systems prepared in acidic media may present higher conductivity due to the increased ionic strength promoted by acetic acid in aqueous environments. Among the isolated systems, PCL/AA exhibited the lowest conductivity, which is consistent with the lower polarity and limited ionization of this polymeric system. Accordingly, the comparatively lower conductivity of A4 among the blank multicomponent formulations is compatible with the incorporation of the low-conductivity PCL phase, although the hydrophilic PVA/gelatin fraction still predominated over the isolated PCL contribution.

The decrease observed after BRPE incorporation suggests that the extract modified the ionic environment of the polymeric solutions, possibly by reducing charge mobility through interactions with polymer chains and/or by changing the balance between conductive and less conductive components in the spinning medium. Since BRPE itself exhibited low conductivity, its incorporation likely contributed minimally as a source of free charge and may have reduced the mobility of ionic species already present in the polymeric systems. This effect can be explained, at least in part, by dilution of ionic species and by possible interactions between phenolic compounds and flavonoids from BRPE and polar groups of the polymers, such as hydroxyl and amide moieties. These interactions may promote intermolecular associations that restrict ionic mobility. A similar trend was reported by Moradkhannejhad et al. [22], who observed reduced electrical conductivity in zein solutions containing increasing concentrations of ethanolic propolis extract.

From an electrospinning perspective, conductivity is directly related to the charge density carried by the solution under the applied electric field and therefore plays a central role in jet stretching and elongation. Higher solution conductivity generally increases charge density on the jet, promotes stronger elongation forces, and tends to produce thinner fibers under comparable processing conditions [31]. Therefore, the reduction in conductivity observed after BRPE incorporation may decrease the electrostatic forces acting on the jet, affecting jet stability and final fiber morphology, including fiber diameter and uniformity. However, conductivity alone is insufficient to predict electrospinning behavior, since fiber formation results from the interplay between electrical forces and the rheological properties of the solution. Consequently, the conductivity data should be interpreted together with the rheological results presented in Section 3.2.2.

#### 3.2.2. Rheological Behavior

**Figure 1:**
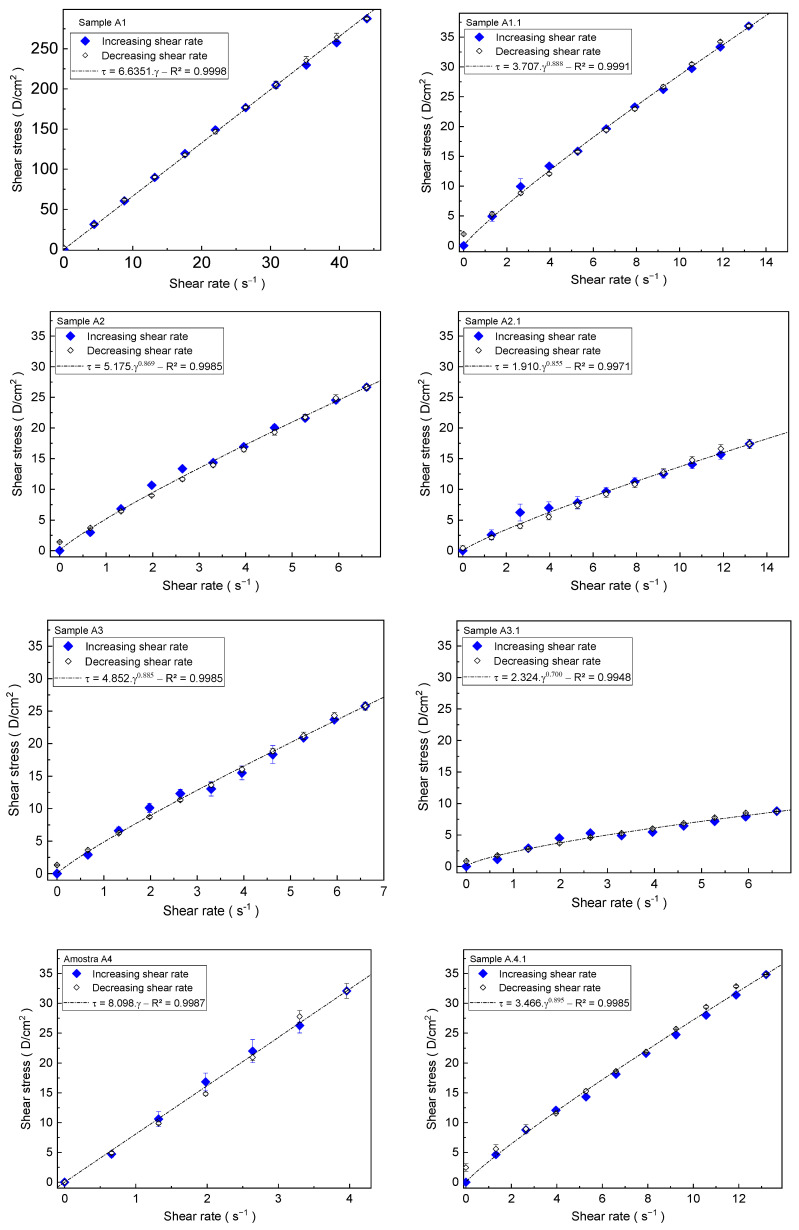

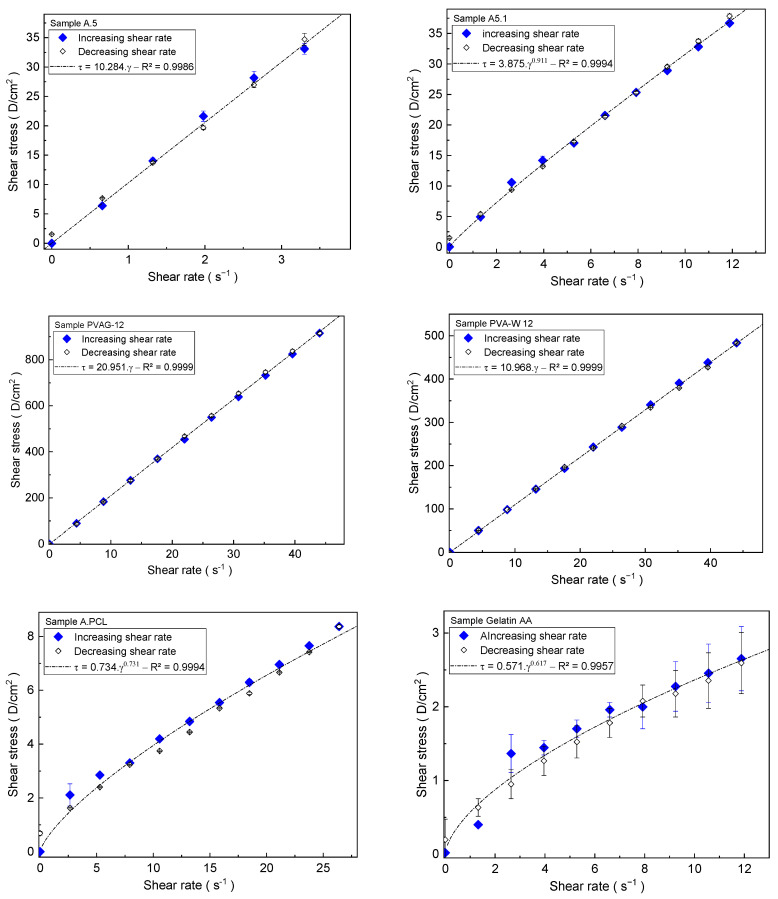
Rheological behavior of the electrospinning formulations with and without BRPE, illustrating the flow profiles used for fitting to the Ostwald–de Waele model.

The rheological analysis showed that the electrospinning formulations exhibited composition-dependent flow behavior, with both Newtonian and pseudoplastic profiles being identified and no evidence of thixotropy (Figure 1). This indicates that the systems remained structurally stable during the measurements and that their flow behavior was primarily governed by formulation composition and shear rate rather than by time-dependent structural breakdown. The experimental data were fitted to the Ostwald–de Waele model (Table 4), with high coefficients of determination (R^2^ = 0.9948–0.9999), confirming the suitability of the power-law model to describe the flow properties of these solutions.

Formulations A1, A4, A5, PVA/G, and PVA/W displayed Newtonian behavior (*n* = 1), indicating nearly constant viscosity throughout the evaluated shear range (Table 4). This behavior is consistent with the predominance of PVA in these systems. PVA is a hydrophilic, flexible, and highly electrospinnable polymer whose hydroxyl-rich chains favor intermolecular hydrogen bonding and chain entanglement, contributing to stable jet formation during electrospinning [31,32]. In the present formulations, the PVA-rich systems maintained sufficient chain mobility and homogeneous flow, which may explain the Newtonian response observed in A1, A4, and A5.

In contrast, formulations A1.1, A2, A2.1, A3, A3.1, A4.1, and A5.1, as well as the isolated gelatin and PCL solutions, showed pseudoplastic behavior (*n* < 1). This shear-thinning profile is typical of polymeric systems in which molecular chains or supramolecular associations progressively align in the direction of flow as shear rate increases. Gelatin contributed to this behavior because of its polypeptide structure and the presence of amide, carboxyl, amino, and hydroxyl groups, which can participate in hydrogen bonding and electrostatic or dipolar interactions with PVA and BRPE constituents. Depending on solvent composition and polymer ratio, these interactions may increase the sensitivity of the blend to shear, favoring pseudoplasticity.

PCL contributed differently to the rheological behavior of the blends. As a more hydrophobic and semicrystalline polyester, PCL has different solvent–polymer interactions compared with PVA and gelatin and may act as a structurally stabilizing component in the final mat. However, in solution, the isolated PCL/AA system showed pseudoplastic behavior, indicating shear-dependent flow. Therefore, the inclusion of PCL in A4/A4.1 may have contributed to the rheological modulation of the multicomponent system, although the hydrophilic PVA/gelatin fraction remained dominant in determining the overall flow behavior of the blend.

The incorporation of BRPE modified the rheological profile of several formulations. In particular, A1.1, A4.1, and A5.1 changed from Newtonian to pseudoplastic behavior after extract incorporation, indicating that the presence of BRPE increased the shear dependence of these systems. This effect may be attributed to transient interactions between phenolic compounds and polymer chains, especially through hydrogen bonding involving hydroxyl, carbonyl, and amide groups. Such interactions may increase intermolecular organization at low shear while allowing progressive alignment or partial disruption of these associations under increasing shear. This interpretation is consistent with electrospinning literature showing that solution composition, molecular interactions, viscosity, polymer concentration, and chain entanglement strongly influence jet stability and fiber formation [31,33,34].

The BRPE-loaded formulations A1.1, A4.1, and A5.1 maintained comparatively higher flow behavior index values than A2.1 and A3.1, indicating more moderate shear-thinning behavior. Conversely, A3.1 showed the lowest *n* value among the loaded blends, suggesting stronger shear dependence and lower resistance to shear-induced flow. These differences indicate that the effect of BRPE was not uniform, but depended on polymer composition, solvent medium, and the balance of extract–polymer interactions within each formulation.

Overall, the rheological results demonstrate that PVA, gelatin, and PCL contributed differently to the flow behavior of the electrospinning solutions. PVA favored homogeneous flow and electrospinnability through chain entanglement and hydrophilicity; gelatin introduced additional functional groups capable of intermolecular interactions and shear-dependent organization; and PCL added a more hydrophobic component with distinct solvent–polymer interactions. BRPE acted as an additional physicochemical modulator, altering the flow behavior of selected blends through probable interactions between phenolic constituents and polymer chains. These effects are relevant for electrospinning because viscosity and chain entanglement must be sufficient to sustain jet continuity, but not so high as to hinder solution flow through the needle [31,35].

Taken together, the conductivity and rheological results show that BRPE affected the electrospinning solutions through combined electrical and viscoelastic mechanisms. The extract reduced conductivity while also increasing shear-dependent behavior in selected formulations. Therefore, the behavior of the BRPE-loaded formulations should be interpreted as the result of a balance between reduced electrical driving force and preserved viscoelastic conditions for spinning, rather than as an isolated effect of conductivity or viscosity alone. This physicochemical characterization provides an important basis for interpreting the subsequent differences in fiber formation, diameter distribution, and structural organization discussed in the following sections.

### 3.3. Electrospinning Performance and Nanofiber Mat Formation

All formulations were successfully processed by electrospinning under the selected conditions, producing continuous nanofibrous mats with sufficient integrity for collection and subsequent characterization. The collected mass varied among formulations, indicating that solution composition influenced deposition efficiency during processing. In general, BRPE-loaded systems yielded comparable or higher collected masses than their respective blank formulations, especially A1.1 and A4.1, suggesting that extract incorporation modified the balance among conductivity, viscosity, and jet stability. These observations are consistent with the electrospinning literature, which identifies solution properties as key determinants of process stability and material formation [31]. Detailed collected-mass data and representative photographs of the mats are provided in the Supplementary Materials (Table S1 and Figure S1). Overall, the selected processing conditions were suitable for the production of BRPE-containing nanofibrous mats, enabling the subsequent evaluation of morphology, porosity, and intermolecular interactions.

### 3.4. Morphological Analysis, Fiber Diameter Distribution, and Surface Porosity of Electrospun Mats

**Figure 2:**
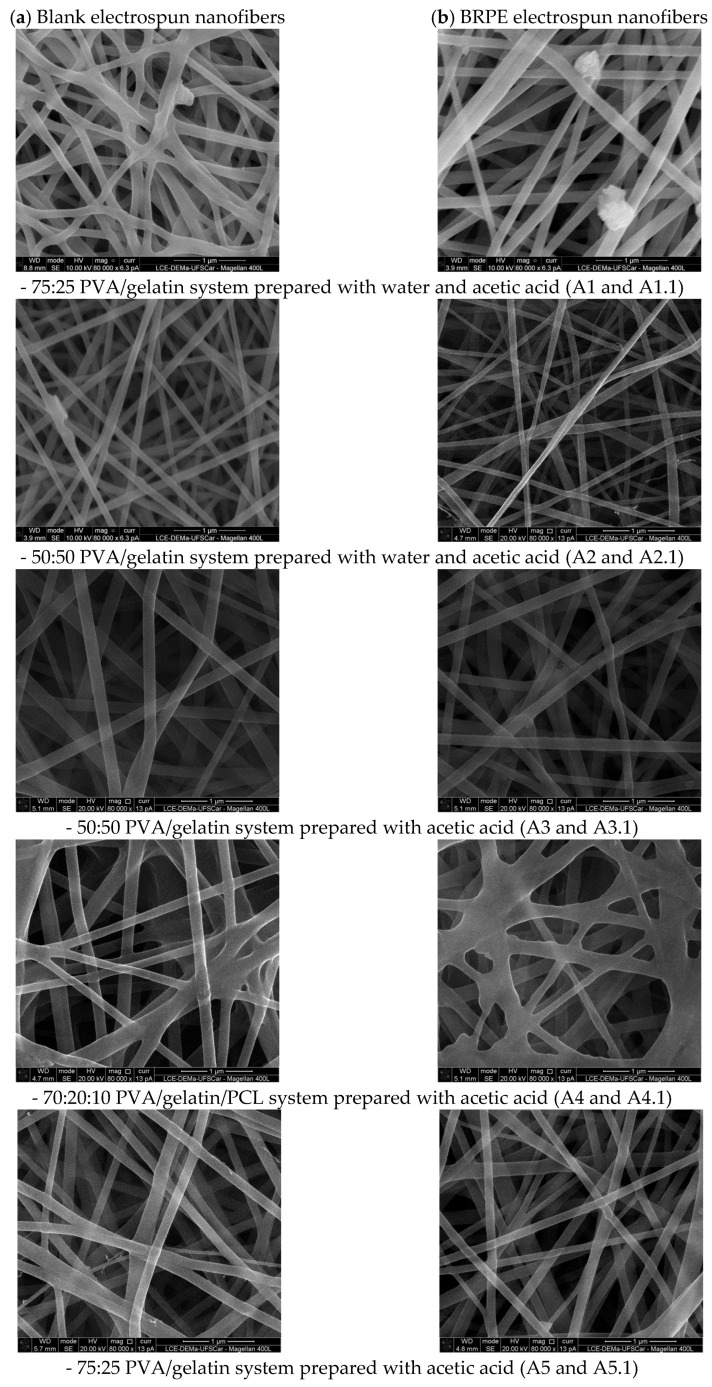
SEM micrographs of blank and BRPE-loaded electrospun nanofibers. Column (**a**) shows blank formulations, and column (**b**) shows the corresponding BRPE-loaded formulations. Formulation codes A1–A5 and A1.1–A5.1 are described in Table 1 and indicated within the figure. BRPE: Brazilian red propolis extract; PVA: poly(vinyl alcohol); PCL: poly(ε-caprolactone).

Scanning electron microscopy (SEM) revealed that all formulations produced continuous nanofibers with smooth morphology and without evident defects such as beads, fused regions, or discontinuities (Figure 2). Overall, the mats exhibited a highly interconnected fibrous network, indicating that the selected processing conditions were suitable for stable electrospinning. This morphological uniformity suggests good compatibility among the solution components and confirms that the systems possessed adequate viscoelastic properties for continuous jet formation, which is consistent with the electrospinning literature for polymer solutions with appropriate conductivity and chain entanglement [31,35].

Quantitative morphological parameters, including mean fiber diameter, standard deviation, D10, D50, D90, and surface porosity, are summarized in Table 5. The mean fiber diameters ranged from 0.094 to 0.224 µm, confirming that both formulation composition and BRPE incorporation affected fiber morphology. In this context, D10 represents the diameter below which 10% of the fibers are distributed, corresponding to the finer fraction of the system; D50 corresponds to the median diameter; and D90 represents the diameter below which 90% of the fibers are found, reflecting the coarser fraction. Despite these variations, all systems maintained good morphological quality, with continuous and structurally well-defined fibers, indicating that BRPE incorporation did not impair spinnability or fiber formation.

The morphological variations observed among the present formulations are consistent with previous reports showing that the effect of propolis-derived bioactives on electrospun fibers is strongly formulation-dependent. In propolis-loaded PVA/PCL systems, Mirbagheri et al. [21] reported submicron fibers in the range of 100–500 nm and showed that propolis affected each polymeric matrix differently: in PVA-based fibers, moderate propolis contents increased the average diameter, whereas excessive loading promoted fiber adhesion; in PCL-based systems, propolis initially reduced fiber diameter, but higher concentrations led to non-uniform and beaded structures. Importantly, their optimized formulations still yielded uniform and bead-free fibers, which supports the interpretation that the incorporation of BRPE, within an adequate compositional window, can modify fiber size without necessarily compromising spinnability.

Chi et al. [14] also showed that the gelatin/PVA ratio strongly affects fiber dimensions, with PVA-rich solutions favoring nanofiber formation due to a suitable balance between viscosity and surface tension, whereas intermediate 50:50 gelatin/PVA compositions produced thicker fibers. Thus, the diameter differences observed here are likely associated with the combined effects of BRPE incorporation and polymer composition, especially the gelatin/PVA balance, which can alter solution properties and jet stretching during electrospinning.

A formulation-dependent response to BRPE incorporation was observed. In some systems, extract loading was associated with an increase in mean fiber diameter, most particularly for A1.1 and A4.1, whereas in others, such as A2.1, A3.1, and A5.1, only modest changes or slight decreases were observed. This behavior indicates that the effect of BRPE on fiber size cannot be attributed to conductivity changes alone, but rather to the combined influence of conductivity, rheological behavior, and intermolecular interactions within each formulation. A similar formulation-dependent effect has been described for propolis-containing electrospun systems, in which changes in solution conductivity and composition influenced final fiber diameter [22]. Accordingly, the present results support the interpretation that fiber morphology was governed by the balance between electrical stretching forces and the viscoelastic resistance of the jet.

A compositional tendency toward morphological stabilization was observed from A3 onward. In particular, A3 appeared to represent a transition point, whereas A4 and A5 defined a more stable region, especially among formulations based on acetic-acid-containing solvent systems. This behavior suggests that, from A3 onward, the systems approached a more favorable balance for homogeneous fiber formation. The diameter descriptors reported in Table 5 support this interpretation and indicate that solvent composition contributed importantly to the solution-to-fiber transition.

Surface porosity estimated from SEM micrographs varied only slightly among the formulations, ranging from 9.8 to 10.6% (Table 5). These values describe the apparent two-dimensional void area visible at the mat surface and should not be interpreted as total porosity, pore volume, or three-dimensional pore-size distribution. Nevertheless, the small variation among formulations indicates that BRPE incorporation did not markedly alter the apparent interfiber surface porosity of the electrospun network. In some extract-loaded pairs, particularly A3/A3.1 and A5/A5.1, a slight reduction in surface porosity was observed, suggesting modestly denser fiber packing after extract incorporation without disruption of the apparent porous architecture of the mats. One possible explanation is that deposition or redistribution of phenolic constituents during solvent evaporation promoted a modest reduction in visible void area within the fibrous network. However, because the analysis was based on SEM images, complementary three-dimensional characterization would be required to confirm changes in the internal porous architecture of the mats.

Although all formulations produced continuous and defect-free nanofibers, the morphological results do not indicate the existence of a single optimized mat. Instead, each polymeric composition contributed differently to the final structure. PVA-rich systems favored electrospinnability and homogeneous fiber formation, probably because of their hydrophilicity, chain entanglement, and adequate viscoelastic behavior [14,31]. Gelatin contributed polar and proteinaceous functional groups that may favor biological interaction, but its effect on fiber size depended strongly on the PVA/gelatin ratio and solvent medium [14]. The inclusion of PCL in A4 introduced a more hydrophobic component and a broader fiber diameter distribution, particularly after BRPE incorporation, as shown by the higher mean diameter and D90 value of A4.1. This interpretation is consistent with the distinct solvent–polymer interactions and hydrophobic character commonly associated with PCL-based electrospun systems. Therefore, the morphological performance should be interpreted as formulation-dependent rather than optimized. From a wound-dressing perspective, fiber continuity and apparent surface porosity were preserved, but these morphological advantages must be balanced against other requirements, including swelling capacity, wet-state structural stability, antimicrobial activity, cytocompatibility, and future mechanical performance.

Overall, the SEM and quantitative morphological data demonstrate that BRPE incorporation modified the solution-to-fiber transition in a formulation-dependent manner while preserving the formation of continuous nanofibrous mats. These findings indicate that the extract influenced microstructural organization without impairing the basic morphological integrity of the electrospun platforms. However, morphology alone does not define an optimized wound-dressing system, and the selection of the most promising formulations must also consider fluid-interaction behavior, wet-state stability, antimicrobial response, cytocompatibility, and future mechanical characterization.

### 3.5. FTIR Analysis and Intermolecular Interactions

**Figure 3:**
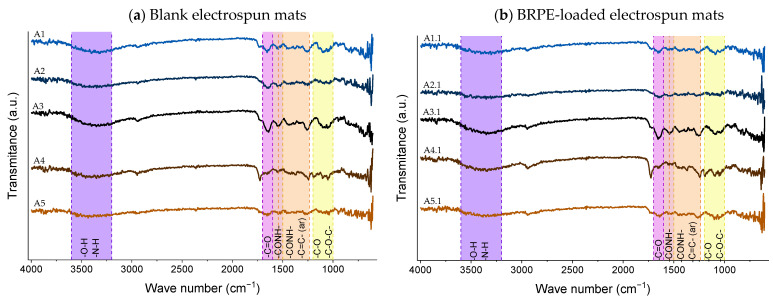
FTIR spectra of blank and BRPE-loaded electrospun mats. Panel (**a**) shows blank electrospun mats, and panel (**b**) shows the corresponding BRPE-loaded mats. Formulation codes A1–A5 and A1.1–A5.1 are described in Table 1. The highlighted regions indicate the main absorption bands associated with O–H/N–H stretching, CH_2_ stretching, PCL carbonyl stretching, amide I, amide II, aromatic C=C vibrations, indicated as C=C-ar in the figure, and C–O/C–O–C vibrations. BRPE: Brazilian red propolis extract; PCL: poly(ε-caprolactone).

FTIR spectra of the electrospun mats showed the presence of the characteristic vibrational bands of the polymeric constituents and revealed subtle spectral changes after incorporation of Brazilian red propolis extract (BRPE), supporting the occurrence of predominantly physical interactions between the extract and the polymer matrix (Figure 3). In all formulations, a broad absorption band was observed in the 3200–3500 cm^−1^ region, which is attributed to O–H and N–H stretching vibrations associated with PVA hydroxyl groups, gelatin functional groups, and phenolic compounds from BRPE. In the BRPE-loaded systems, changes in band shape and intensity in this region suggest modification of the hydrogen-bonding environment within the matrix. This interpretation is consistent with previous reports on electrospun PVA/gelatin systems and phenolic plant extracts analyzed by FTIR, in which changes in the O–H/N–H stretching region have been associated with non-covalent interactions involving hydroxyl-containing compounds [36,37].

In the 1650–1450 cm^−1^ region, the spectra displayed bands associated with amide I and amide II vibrations of gelatin. Samples containing BRPE also showed spectral contributions in this region that are compatible with overlapping aromatic C=C vibrations from flavonoids and other phenolic constituents of red propolis. This interpretation is reasonable considering the chemically complex phenolic profile reported for Brazilian red propolis, including flavonoids and isoflavonoids that contribute to its antioxidant and biological properties [17]. Although these spectral changes were not accompanied by the appearance of new isolated bands, they suggest that BRPE modified the local molecular environment of the gelatin-containing matrix.

Additional differences were observed in the 1200–1000 cm^−1^ fingerprint region, where BRPE-loaded mats displayed bands associated with phenolic C–O stretching and C–O–C vibrations of flavonoids and related compounds. These changes reflect the spectral complexity of the extract and provide further evidence of its incorporation into the nanofibrous system [37]. For formulations containing PCL (A4 and A4.1), characteristic absorptions near 2950 cm^−1^ and 1720–1730 cm^−1^ were also observed, corresponding respectively to asymmetric CH_2_ stretching and carbonyl (C=O) stretching of PCL. These bands partially overlapped with absorptions from the gelatin-containing matrix, as expected for multicomponent electrospun systems containing both polyester and protein fractions [38].

Overall, the FTIR spectra did not provide evidence of new covalent bond formation after BRPE incorporation. Instead, the observed band-shape changes, intensity variations, and increased spectral complexity are more consistent with non-covalent interactions, especially hydrogen bonding and packing effects, between BRPE constituents and the polymeric network. This interpretation is in agreement with the preserved fibrous morphology observed by SEM and with the modest changes in fiber diameter and surface porosity. Therefore, FTIR supports the view that BRPE was incorporated into the electrospun matrices mainly through physical association with the polymer chains, without disrupting the overall structural organization of the nanofibers.

### 3.6. HPLC-DAD Characterization of BRPE and BRPE-Loaded Electrospun Nanofibers

HPLC-DAD analysis was performed to characterize BRPE and to verify whether characteristic phenolic marker peaks remained detectable after incorporation into the electrospun nanofibers. This analysis was particularly relevant because Brazilian red propolis is known to contain a chemically complex and variable phenolic profile rich in flavonoids, isoflavonoids, and related compounds, and its standardization requires reliable analytical approaches [17,18,39]. Previous studies have identified liquiritigenin and formononetin among the characteristic constituents of this propolis type, together with calycosin, isoliquiritigenin, vestitol, neovestitol, medicarpin, biochanin A, and 7-O-methylvestitol [17,18]. In the present work, LQ and FN were selected as representative marker peaks for qualitative and quantitative monitoring after electrospinning.

The HPLC-DAD method showed suitable linearity within the evaluated concentration range (1–100 µg/mL), with coefficients of determination of 0.9981 for LQ and 0.9992 for FN, confirming the adequacy of the method for quantitative analysis of the selected markers. This is consistent with previous studies showing that HPLC-DAD is a suitable and accessible tool for phenolic standardization and quality control of complex natural products, including propolis and plant-derived extracts [17,29,39].

The chromatographic profile of BRPE recorded at 281 nm showed two major peaks at retention times of 13.041 min and 24.797 min. The first peak was assigned to liquiritigenin (LQ) based on comparison with the authentic standard, showing agreement in both retention time and UV spectrum. The second peak, at 24.797 min, was treated as the formononetin (FN)-associated marker peak, since its retention time was compatible with that of the authentic standard and peak-purity analysis indicated a highly homogeneous signal. Although the DAD spectrum of the second extract peak was not fully superimposable on that of the pure standard, this difference did not preclude its use for comparative fingerprinting and quantitative monitoring under the analytical conditions employed.

The same two marker peaks were detected in all BRPE-loaded nanofibers, with only minor variation in retention times: A1.1, 13.106 and 24.858 min; A2.1, 13.136 and 24.860 min; A3.1, 13.094 and 24.824 min; A4.1, 13.063 and 24.811 min; and A5.1, 13.069 and 24.789 min. The close similarity among these chromatographic profiles indicates that electrospinning preserved the characteristic BRPE fingerprint after processing. This observation agrees with previous reports on propolis-containing electrospun systems, in which the extract could be incorporated into fibrous matrices while maintaining detectable chemical signatures and biological activity [22]. Representative HPLC-DAD chromatograms of BRPE and BRPE-loaded electrospun nanofibers, together with the analytical standards and UV spectra used for peak assignment, are provided in the Supplementary Materials (Figure S2).

Quantitative analysis confirmed the presence of both marker peaks in all BRPE-loaded formulations. LQ contents ranged from 0.113 ± 0.005 to 0.142 ± 0.001% (d.b.), corresponding to retention efficiencies between 80 ± 3% and 101 ± 1%. FN-marker contents ranged from 0.638 ± 0.030 to 0.816 ± 0.006% (d.b.), corresponding to retention efficiencies between 76 ± 4% and 98 ± 1%. A3.1 showed the highest retention for both marker compounds, with 101 ± 1% for LQ and 98 ± 1% for the FN-associated peak, indicating essentially complete retention within experimental variability. In contrast, A4.1 showed the lowest efficiencies, with 80 ± 3% for LQ and 76 ± 4% for the FN-associated peak. The remaining formulations exhibited consistently high retention, with LQ efficiencies between 93 ± 2% and 95 ± 7% and FN efficiencies between 91 ± 1% and 93 ± 2%. Detailed replicate values used for marker quantification and retention-efficiency calculations are provided in the Supplementary Materials (Table S3).

The observed differences among the formulations suggest that the polymer composition and solvent system influenced the incorporation and/or extractability of BRPE constituents within the nanofibrous matrices. In general, LQ showed more homogeneous retention across the formulations, whereas the FN-associated peak exhibited slightly greater variation. Because the systems differed in PVA/gelatin ratio, solvent composition, and the presence or absence of PCL, these results likely reflect formulation-dependent interactions between BRPE constituents and the polymer network during solution preparation and solvent evaporation. Similar formulation-dependent behavior has been reported for propolis-containing electrospun systems, in which the nature of the polymer matrix influenced the morphology, extract distribution, and performance of the final material [22]. Even so, no major qualitative loss of the chromatographic profile was observed in any of the loaded systems.

Overall, the HPLC-DAD results demonstrate that BRPE was successfully incorporated into the electrospun nanofibers while preserving the main chromatographic features of the native extract and retaining substantial amounts of characteristic phenolic marker signals after processing. The assignment of the LQ peak was supported by agreement in retention time and UV spectrum with the authentic standard. For FN, the assignment was considered tentative, since retention time and peak-purity analysis supported the attribution, although the UV spectrum of the extract peak did not fully coincide with that of the pure standard. Thus, the present HPLC-DAD analysis supports the use of these peaks as characteristic marker signals for comparative and quantitative monitoring of BRPE and BRPE-loaded nanofibers under the analytical conditions employed.

### 3.7. Fluid Interaction Behavior Under Simulated Wound Conditions

#### Swelling and Coupled Degradation Behavior

The fluid interaction behavior of the electrospun mats was evaluated in two simulated wound media, Solution A and simulated wound fluid A (SWF A), in order to investigate how the formulations responded under exudate-like conditions. This type of assessment is relevant for wound dressing development because the composition and physicochemical properties of simulated wound fluids directly influence the measured fluid-handling performance of a dressing, including swelling, retention, and structural preservation [24]. The pH and electrical conductivity of the simulated wound media used in the fluid interaction studies are shown in Table 6. In practical terms, an effective wound dressing should absorb exudate while maintaining sufficient structural integrity to preserve coverage of the wound bed and avoid premature disintegration [7,40].

In the present study, the highly hydrophilic character of the PVA/gelatin-rich matrices strongly influenced their response after contact with the simulated wound fluids. Samples A1, A2, and A3 showed rapid structural collapse during the first evaluation interval, preventing reliable gravimetric monitoring. This behavior indicates that, although these formulations interacted readily with aqueous media, their stability under simulated wound conditions was limited. Such a response is consistent with the known tendency of gelatin- and PVA-rich systems to absorb water rapidly and undergo pronounced swelling and erosion unless adequate stabilization is introduced into the fibrous network [41,42]. Therefore, in these systems, fluid uptake appeared to be closely coupled to matrix disintegration.

The swelling-associated degradation behavior of the electrospun mats in Solution A and SWF A is summarized in Table 7. Because several formulations underwent rapid structural collapse during the first evaluation interval, only samples that maintained sufficient integrity for reliable gravimetric quantification are reported. The values are presented descriptively, and no inferential statistical analysis was performed because replicate-based variability estimates were not available for these measurements.

The A5 electrospun nanofiber mat showed the lowest mass loss in both media, with 35% in Solution A and 39% in SWF A, indicating the most favorable structural preservation under wet conditions. In contrast, the BRPE-loaded formulations showed higher mass loss, ranging from 47% to 64% in Solution A and from 54% to 70% in SWF A. SWF A consistently produced slightly higher mass loss than Solution A, suggesting that the more complex simulated wound medium intensified matrix erosion, probably due to its higher ionic strength and protein-containing composition. Overall, these results suggest that the 75:25 PVA/gelatin formulation prepared in an acetic-acid-containing medium provided a more suitable compromise between hydrophilicity and network cohesion than the other highly hydrophilic systems. From a pharmaceutical perspective, this balance is relevant because wound-dressing materials should interact with exudate while maintaining sufficient structural integrity under wet conditions.

In contrast, the PCL-containing formulations A4 and A4.1 did not maintain structural integrity under the same conditions, indicating that the presence of PCL at the tested proportion was not sufficient to overcome the destabilizing effects imposed by the overall blend composition and fluid–matrix interactions. This finding reinforces that fluid response in multicomponent electrospun mats depends not only on the presence of a more hydrophobic polymer, but also on its relative amount, distribution within the fibers, and interaction with the more hydrophilic phases of the system [7,41].

The direct comparison between A5 and A5.1 is particularly informative because both formulations share the same PVA/gelatin ratio and acetic-acid-based solvent system, differing mainly by the presence of BRPE. In this pair, BRPE incorporation increased mass loss from 35% to 64% in Solution A and from 39% to 70% in SWF A. Thus, under the tested conditions, BRPE did not act as a structural stabilizer of the wet mat. Instead, its incorporation may have increased matrix hydration, plasticization, or disruption of polymer–polymer interactions, thereby facilitating erosion in aqueous media.

These findings indicate that the incorporation of BRPE can modulate the biological functionality of electrospun mats, but does not necessarily improve their wet-state structural stability. Therefore, future formulation development should focus on strategies to improve matrix cohesion, such as controlled crosslinking, bilayer architectures, or adjustment of the hydrophilic/hydrophobic polymer balance. In this context, A5 provides a useful structural reference formulation, whereas BRPE-loaded systems require further optimization to combine bioactivity with adequate resistance to degradation under simulated wound conditions. Overall, the fluid-response data reinforce that future formulation optimization should focus on increasing matrix stability while preserving sufficient hydrophilicity for exudate uptake, since both properties are essential for pharmaceutical performance in wound care [7,40].

### 3.8. Biological Evaluation of Electrospun Mats

#### 3.8.1. Antimicrobial Activity

The antimicrobial activity of the BRPE-loaded electrospun mats was evaluated by agar diffusion as a first-stage screening assay, an approach commonly used for preliminary assessment of antimicrobial wound dressings and plant-derived antimicrobial systems [43,44]. This method was selected because the antimicrobial performance was assessed directly from the solid electrospun mats, rather than from a soluble extract or isolated antimicrobial compound. Therefore, the assay provides an initial indication of whether bioactive constituents incorporated into the fibrous matrix can diffuse into the agar medium and inhibit microbial growth around the sample. The measured inhibition-zone diameters represent estimates of the diffusion-dependent antimicrobial response of the solid mats and should not be interpreted as direct measures of antimicrobial potency. In addition, this assay does not provide MIC or MBC values and does not distinguish bacteriostatic from bactericidal effects.

The BRPE-loaded electrospun mats showed a microorganism-dependent antimicrobial profile, indicating that the biological activity of the extract was at least partially preserved after incorporation into the nanofibrous systems, although not uniformly across all tested strains (Table 8). The most pronounced responses were observed against *Staphylococcus epidermidis*, particularly for formulations A1.1 and A3.1, while *Klebsiella pneumoniae* showed consistent inhibitory activity across all formulations. No inhibition was detected against *Pseudomonas aeruginosa*. Partial or formulation-dependent effects were observed for *Escherichia coli*, *Enterococcus faecalis*, *Candida albicans*, and *Staphylococcus aureus*. This pattern is consistent with the known antimicrobial behavior of propolis-derived phenolic compounds, which often show stronger activity against some Gram-positive species while displaying more limited activity against intrinsically resistant Gram-negative pathogens [22,45].

The activity observed against *S. epidermidis* is particularly relevant from a wound-care perspective, since this species is frequently associated with colonized wounds and biomaterial-related contamination. The consistent inhibitory response against *K. pneumoniae* deserves attention because this microorganism is increasingly recognized in acute and chronic wound infections and is associated with biofilm formation and antimicrobial resistance. In contrast, the absence of activity against *P. aeruginosa* was not unexpected, considering the intrinsic resistance of this pathogen, which is associated with its outer membrane barrier, low permeability, and multiple efflux mechanisms. Therefore, the present mats should not be interpreted as broad-spectrum antimicrobial dressings, but rather as materials with selective antimicrobial potential against clinically relevant wound-associated microorganisms [45,46].

The formulation-dependent antimicrobial response suggests that polymer composition and matrix hydration influenced the availability of BRPE constituents during the agar diffusion assay. More hydrophilic PVA/gelatin-rich matrices may hydrate rapidly upon contact with agar, favoring partial release of phenolic constituents into the surrounding medium. Conversely, the presence of PCL in A4.1 may have reduced matrix hydration and limited diffusion of BRPE-associated compounds, generally weaker or absent inhibition observed for this formulation in several tested strains despite identical extract loading. Therefore, antimicrobial performance appears to depend not only on BRPE content, but also on the balance among polymer composition, matrix hydration, and diffusion of active constituents.

The partial activity observed against selected strains, including *C. albicans* only in formulation A2.1, suggests that antimicrobial action may also have been influenced by the physical behavior of the mats during the agar diffusion assay. Because electrospun mats can partially hydrate, soften, or form a gel-like interface upon contact with moist agar, diffusion of active constituents into the culture medium may be restricted. Under such conditions, agar diffusion may underestimate the biological effect of fiber-incorporated actives, especially when the bioactive is retained within the polymeric network rather than rapidly released into the surrounding medium. This limitation has also been discussed for propolis-containing fibrous systems and for electrospun wound dressings more broadly [22,47].

Overall, the antimicrobial results support the potential of BRPE-loaded nanofibers as adjunct bioactive wound-dressing platforms, particularly where partial control of susceptible Gram-positive bacteria and selected opportunistic pathogens is desirable. At the same time, the lack of activity against *P. aeruginosa* and the variable responses among formulations and microorganisms indicate that antimicrobial performance remains formulation-dependent and incomplete. Thus, from a pharmaceutical development standpoint, these materials are promising but still require optimization, particularly with respect to extract loading, matrix composition, and the relationship between matrix hydration and bioactive availability [46,48]. Future studies should include release assays, MIC and MBC determinations using solubilized BRPE or mat extracts, time-kill studies, and antibiofilm assays to better define the antimicrobial potency and mechanism of action of these systems.

#### 3.8.2. Cytocompatibility in HaCaT Cells

**Figure 4:**
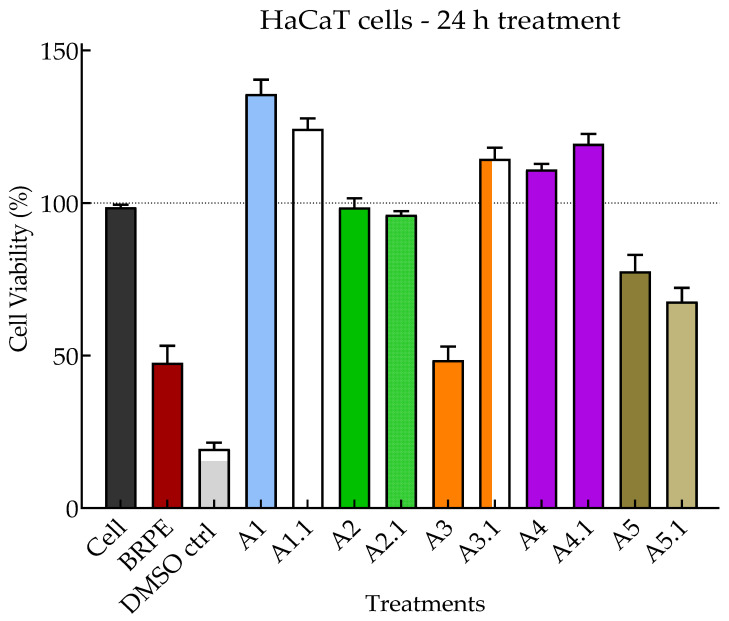
Cell viability of HaCaT cells after 24 h exposure to free BRPE, controls, and electrospun mats, as determined by the MTT assay. Formulation pairs are represented by related color tones to facilitate comparison between blank and BRPE-loaded systems. The dashed horizontal line indicates 100% viability relative to untreated control cells. Data are expressed as mean ± standard deviation. BRPE: Brazilian red propolis extract; DMSO: dimethyl sulfoxide.

The MTT assay showed that the cytocompatibility of the system depended strongly on whether BRPE was tested in its free form or incorporated into the electrospun matrices (Figure 4). Free BRPE showed marked cytotoxicity, with cell viability around 50%, indicating that direct exposure to the native extract at the tested condition was unfavorable for keratinocyte survival. This result is plausible considering the complex phenolic composition of Brazilian red propolis, whose bioactive constituents may be beneficial at controlled exposure levels but may also become deleterious when presented in a concentrated, unmodulated form [45,47].

After incorporation into the electrospun matrices, however, the biological response changed markedly and became formulation-dependent. Selected BRPE-loaded mats, especially A1.1 and A3.1, showed improved cytocompatibility, reaching approximately 138% and 127% viability, respectively. These results suggest that the polymeric nanofibrous matrix modulated cellular exposure to the extract and reduced the adverse effect observed for the free form. Importantly, this interpretation should be made cautiously: the present data are consistent with matrix-mediated modulation of BRPE availability, but they do not by themselves demonstrate a defined release mechanism. Direct release studies would be required to confirm whether the improved cytocompatibility is driven by slower release, lower local concentration, altered extract presentation, or a combination of these factors.

The behavior of the formulations also indicates that cytocompatibility was not determined by BRPE alone, but by the interaction between extract incorporation and matrix composition. The PVA/gelatin blank systems were already favorable for cell survival, whereas extract incorporation produced different outcomes depending on the formulation. In A1.1 and A3.1, BRPE incorporation was associated with improved viability, which may reflect a better balance between antioxidant bioactivity and matrix-mediated moderation of exposure. In contrast, formulations such as A2.1 and A5.1 showed less favorable behavior, indicating that small compositional differences were sufficient to alter the biological response. This formulation dependence is highly relevant for pharmaceutical development because it shows that the same extract can shift from beneficial to unfavorable depending on the architecture and composition of the fibrous carrier [46,48].

From the perspective of wound-dressing applications, these findings are encouraging because keratinocyte compatibility is essential for re-epithelialization and tissue repair. The present data suggest that electrospinning can serve not only as a fabrication method, but also as a means of modulating how a chemically complex natural extract interacts with cells. At the same time, the variability among formulations shows that cytocompatibility cannot be assumed simply from extract incorporation. Instead, it must be optimized at the level of the polymer–bioactive system as a whole. Accordingly, the present manuscript supports the concept of matrix-dependent biological modulation, but future studies should include direct release assessment and longer-term cell-response analyses to clarify the mechanisms underlying the observed differences [45,46].

## 4. General Discussion and Pharmaceutical Implications

The present study shows that BRPE incorporation influenced the electrospun systems at multiple and interconnected levels, from solution behavior and fiber formation to fluid interaction, antimicrobial activity, cytocompatibility, and preservation of characteristic chemical marker signals. These findings indicate that electrospinning did not merely entrap the extract in a passive carrier, but generated formulation-dependent changes in the physicochemical and biological behavior of the mats. In this context, the electrospun matrix should be viewed as an active modulator of BRPE performance rather than simply as a support material.

At the same time, the results show that no single formulation simultaneously optimized all the properties desirable for wound-dressing applications. The more hydrophilic systems interacted rapidly with aqueous media but lacked sufficient structural stability under simulated wound conditions, whereas more favorable biological responses were observed only in selected BRPE-loaded formulations. Likewise, antimicrobial activity was selective rather than broad-spectrum, and the absence of activity against *P. aeruginosa* remains an important limitation for infected-wound applications. In addition, tensile properties and surface roughness were not evaluated in the present study. These parameters are important for wound-dressing development because mechanical strength, elongation, and wet-state stability influence handling, application, and performance in contact with wound exudate, whereas surface roughness may affect cell adhesion, fluid interaction, and biological response. Future studies should therefore include tensile strength, elongation at break, Young’s modulus, wet-state mechanical stability, and AFM- or profilometry-based roughness analysis. Porosity characterization was also limited to two-dimensional SEM micrographs. Although this approach is useful for preliminary comparison of the apparent interfiber surface porosity of electrospun mats, it does not provide total porosity, pore volume, or three-dimensional pore-size distribution. Future studies should therefore include complementary techniques such as BET analysis, mercury intrusion porosimetry, or micro-CT to better characterize the internal porous architecture of the mats. Thus, the pharmaceutical potential of BRPE-loaded electrospun mats is clear, but still depends on further optimization of structural stability, mechanical performance, fluid handling, porosity characterization, surface properties, and bioactive availability.

From a development perspective, the main advance of this work is the demonstration that native Brazilian red propolis extract can be incorporated into electrospun polymeric nanofibers while preserving characteristic chemical markers and biologically relevant activity and improving cytocompatibility in selected systems. The principal limitation is that this incorporation does not automatically result in an optimal wound dressing, since performance remains strongly dependent on the balance among polymer composition, matrix organization, hydrophilicity, and extract–matrix interactions. Overall, the study provides a solid proof of concept and a relevant basis for further formulation optimization toward pharmaceutical application.

## 5. Conclusions

Electrospinning was shown to be a viable strategy for incorporating Brazilian red propolis extract (BRPE) into polymeric nanofibers and generating wound-dressing candidates with formulation-dependent physicochemical and biological performance. BRPE incorporation modified solution conductivity and rheological behavior, which influenced fiber morphology and, consequently, fluid interaction, antimicrobial response, and cytocompatibility. Selected BRPE-loaded formulations showed promising biological performance, including selective antimicrobial activity and improved HaCaT cell viability, whereas the more hydrophilic systems exhibited rapid fluid uptake but limited structural stability under simulated wound conditions. These findings support the potential of BRPE-loaded electrospun mats as multifunctional wound-dressing platforms while also showing that further optimization is required to improve structural stability and better modulate bioactive availability. Further studies should include mechanical testing, wet-state stability assessment, surface roughness analysis, release kinetics, and in vivo wound-healing evaluation to advance these proof-of-concept nanofibrous platforms toward pharmaceutical application.

## Figures and Tables

**Table 1:** Composition of simulated wound fluid A (SWF A) and Solution A.

	Components	SWF A	Solution A
Salts	Sodium chloride (NaCl)	110 mM	8.298 g/L
	Calcium chloride (CaCl_2_)	12.2 mM	0.368 g/L
	Potassium chloride (KCl)	2.7 mM	-
	Magnesium chloride (MgCl_2_)	0.5 mM	-
Protein	BSA	34 g/L	-
Buffers	Potassium phosphate (KH_2_PO_4_)	1.3 mM	-
	Sodium bicarbonate (NaHCO_3_)	20 mM	-

**Table 2:** Composition of electrospun polymeric solutions.

Sample	PVA 12%, Solvent	Gelatin 12%, Solvent	PCL 20%, Solvent	Polymers Ratio (*v*/*v*)	BRPE (% *w*/*w*)
A1	W	AA 90%	-	75:25 PVA:Gel	
A1.1	W	AA 90%	-	75:25 PVA:Gel	10
A2	W	AA 90%	-	50:50 PVA:Gel	
A2.1	W	AA 90%	-	50:50 PVA:Gel	10
A3	AA 70%	AA 90%	-	50:50 PVA:Gel	
A3.1	AA 70%	AA 90%	-	50:50 PVA:Gel	10
A4	AA 70%	AA 90%	AA 90%	70:20:10 PVA:Gel:PCL	
A4.1	AA 70%	AA 90%	AA 90%	70:20:10 PVA:Gel:PCL	10
A5	AA 70%	AA 90%	-	75:25 PVA:Gel	
A5.1	AA 70%	AA 90%	-	75:25 PVA:Gel	10

AA: aqueous glacial acetic acid; W: water; *v*/*v*: volume ratio; % *w*/*w*: weight percentage.

**Table 3:** Electrical conductivity of blank and BRPE-loaded electrospinning formulations.

Pair	Composition	Blank (µS cm^−1^)	BRPE-Loaded (µS cm^−1^)	Reduction (%)
A1/A1.1	75:25 PVA/W:Gel/AA	1586.0 ± 7.8	700.8 ± 0.4 ***	55.8
A2/A2.1	50:50 PVA/W:Gel/AA	1502.5 ± 1.6	779.6 ± 0.1 ***	48.1
A3/A3.1	50:50 PVA/AA:Gel/AA	1102.1 ± 11.2	705.2 ± 2.5 ***	36.0
A4/A4.1	70:20:10 PVA/AA:Gel/AA:PCL/AA	919.6 ± 0.6	539.6 ± 3.2 ***	41.3
A5/A5.1	75:25 PVA/AA:Gel/AA	1073.3 ± 1.3	608.0 ± 0.2 ***	43.3

Values are expressed as mean ± SD (*n* = 3). Conductivity reduction was calculated relative to the corresponding blank formulation. *** *p* < 0.001 vs. the corresponding blank formulation (Student’s *t*-test). AA: acetic acid medium; W: water; BRPE: Brazilian red propolis extract; PVA: poly(vinyl alcohol); PCL: poly(ε-caprolactone); Gel: gelatin.

**Table 4:** Ostwald–de Waele model parameters and coefficients of determination (R^2^) for the electrospinning formulations.

Samples	µ * (P)	K (P·s^n−1^)	*n* (-)	R^2^ (-)
A1	6.635	-	1.00	0.9998
A1.1	-	3.707	0.888	0.9991
A2	-	5.175	0.869	0.9985
A2.1	-	1.910	0.855	0.9971
A3	-	4.852	0.885	0.9985
A3.1	-	2.324	0.700	0.9948
A4	8.098	-	1.00	0.9987
A4.1	-	3.466	0.895	0.9985
A5	10.284	-	1.00	0.9986
A5.1	-	3.875	0.911	0.9994
PVAG-12	20.951	-	1.00	0.9999
PVAW-12	10.968	-	1.00	0.9999
PCL AA	-	0.734	0.731	0.9994
Gelatin AA	^-^	0.571	0.617	0.9957

µ * = Viscosity of Newtonian formulations; K: consistency index; *n*: flow behavior index; R^2^: coefficient of determination.

**Table 5:** Mean fiber diameter, standard deviation, diameter descriptors (D10, D50, and D90), and surface porosity of the electrospun nanofibrous mats.

Sample	Mean Diameter (µm)	D10 (µm)	D50 (µm)	D90 (µm)	Porosity (%)
A1	0.130 ± 0.040	0.0930	0.1200	0.1760	9.8
A1.1	0.165 ± 0.043	0.1259	0.1565	0.2103	9.8
A2	0.119 ± 0.030	0.0878	0.1125	0.1531	10.2
A2.1	0.094 ± 0.025	0.0670	0.0880	0.1344	9.9
A3	0.168 ± 0.037	0.1148	0.1710	0.2175	10.6
A3.1	0.161 ± 0.047	0.1209	0.1485	0.2226	10.3
A4	0.176 ± 0.054	0.1227	0.1655	0.2530	10.5
A4.1	0.224 ± 0.120	0.1166	0.1880	0.4298	10.4
A5	0.147 ± 0.033	0.1148	0.1410	0.1853	10.5
A5.1	0.140 ± 0.048	0.0907	0.1310	0.2043	10.2

**Table 6:** Physicochemical properties of the simulated wound media used for fluid interaction studies.

Medium	pH	Electrical Conductivity (µS/cm)
Solution A	6.78	10,624.25
SWF A	7.27	13,835.00

**Table 7:** Mass loss of electrospun mats after exposure to simulated wound media.

Formulation	BRPE Loading	Solution A Mass Loss (%)	SWF A Mass Loss (%)	Structural Interpretation
A1.1	10% *w*/*w*	47	54	Partial preservation
A2.1	10% *w*/*w*	59	63	Pronounced degradation
A3.1	10% *w*/*w*	60	64	Pronounced degradation
A5.1	10% *w*/*w*	64	70	Pronounced degradation
A5	No BRPE	35	39	Best preservation

Data are presented as descriptive mass-loss values after exposure to Solution A and simulated wound fluid A (SWF A). BRPE: Brazilian red propolis extract. Formulations A1, A2, A3, A4, and A4.1 underwent rapid structural collapse during the first evaluation interval, preventing reliable gravimetric quantification.

**Table 8:** Antimicrobial activity of BRPE-loaded electrospun nanofibers against clinically relevant microorganisms, expressed qualitatively and by inhibition-zone diameter (ZOI).

	Samples	A1.1	A2.1	A3.1	A4.1	A5.1
Microorganisms						
*P. aeruginosa*		No activity	No activity	No activity	No activity	No activity
*E. faecalis*		Partial halo (8.4 ± 0.1 mm) 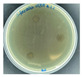	ND (*n* = 2) 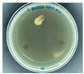	No activity	No activity	ND (*n* = 1) 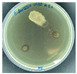
*E. coli*		Partial halo (8.6 ± 0.3 mm) 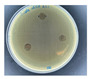	Partial halo (8.5 ± 0.3 mm) 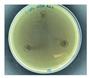	Partial halo (8.5 ± 0.2 mm) 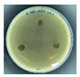	No activity	Partial halo (8.6 ± 0.2 mm) 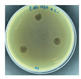
*S. aureus*		No activity	Partial halo (8.5 ± 0.2 mm) 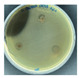	No activity	No activity	No activity
*S. epidermidis*		Activity (8.7 ± 0.2 mm) 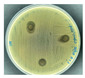	Partial halo (8.6 ± 0.2 mm) 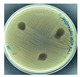	Activity (9.5 ± 0.2 mm) 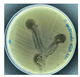	ND (*n* = 2) 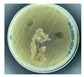	Partial halo (8.6 ± 0.2 mm) 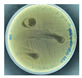
*Candida albicans*		No activity	Partial halo (10.1 ± 0.5 mm) 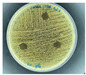	No activity	No activity	No activity
*Klebsiella pneumoniae*		Activity (9.6 ± 0.9 mm) 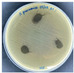	Activity (9.9 ± 0.7 mm) 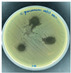	Activity (10.2 ± 0.7 mm) 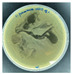	Partial halo (8.1 ± 0.4 mm) 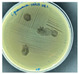	Activity (9.2 ± 0.6 mm) 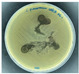

Values are expressed as mean ± SD (*n* = 3 valid measurements). Activity: clear inhibition zone without visible growth; Partial halo: diffuse inhibition zone with reduced growth; No activity: absence of a measurable zone (mean/SD not applicable). ND (not determined): fewer than three valid ZOI measurements available. Contaminated or irregular measurements were excluded from calculations.

## Data Availability

The data presented in this study are available from the corresponding author upon reasonable request.

## Supplementary Materials

The following supporting information can be downloaded at: https://www.mdpi.com/article/10.3390/pharmaceutics18070888/s1. Table S1. Mass of electrospun nanofibrous mats collected on the aluminum support after processing. Table S2. Summary of HPLC-DAD quantification and retention efficiency of liquiritigenin (LQ) and the formononetin-associated marker signal (FN) in BRPE-loaded electrospun nanofibers. Table S3. Replicate analytical values used for marker quantification and retention-efficiency calculations. Figure S1. Representative photographs of the electrospun nanofibrous mats deposited on the aluminum support after electrospinning, illustrating their visual integrity and collectability. Figure S2. Representative HPLC-DAD chromatograms recorded at 281 nm and UV spectra used for qualitative confirmation of BRPE marker signals. Figure S3. Representative HPLC-DAD chromatograms recorded at 281 nm and UV spectra used for qualitative confirmation of BRPE marker signals in the electrospun nanofibers.
