# Supplementary material for: Electrospun Polymeric Nanofibers Incorporating Brazilian Red Propolis Extract for Wound Dressing Applications
Source: Pharmaceutics. 2026 Jul 20;18(7):888. doi: 10.3390/pharmaceutics18070888 (PMC13414978; doi:10.3390/pharmaceutics18070888)
Supplement: Supplementary file 1 [file pharmaceutics-18-00888-s001.zip › pharmaceutics-4349290-Supplementary S1.pdf]

pharmaceutics

MDPI

Supplementary Material S1

Table S1. Mass of electrospun nanofibrous mats collected on the aluminum support after processing.

Sample	Mass (mg)	Sample	Mass (mg)
A1	27.80	A1.1	96.10
A2	153.40	A2.1	126.50
A3	142.50	A3.1	123.90
A4	39.80	A4.1	100.50
A5	138.00	A5.1	105.40

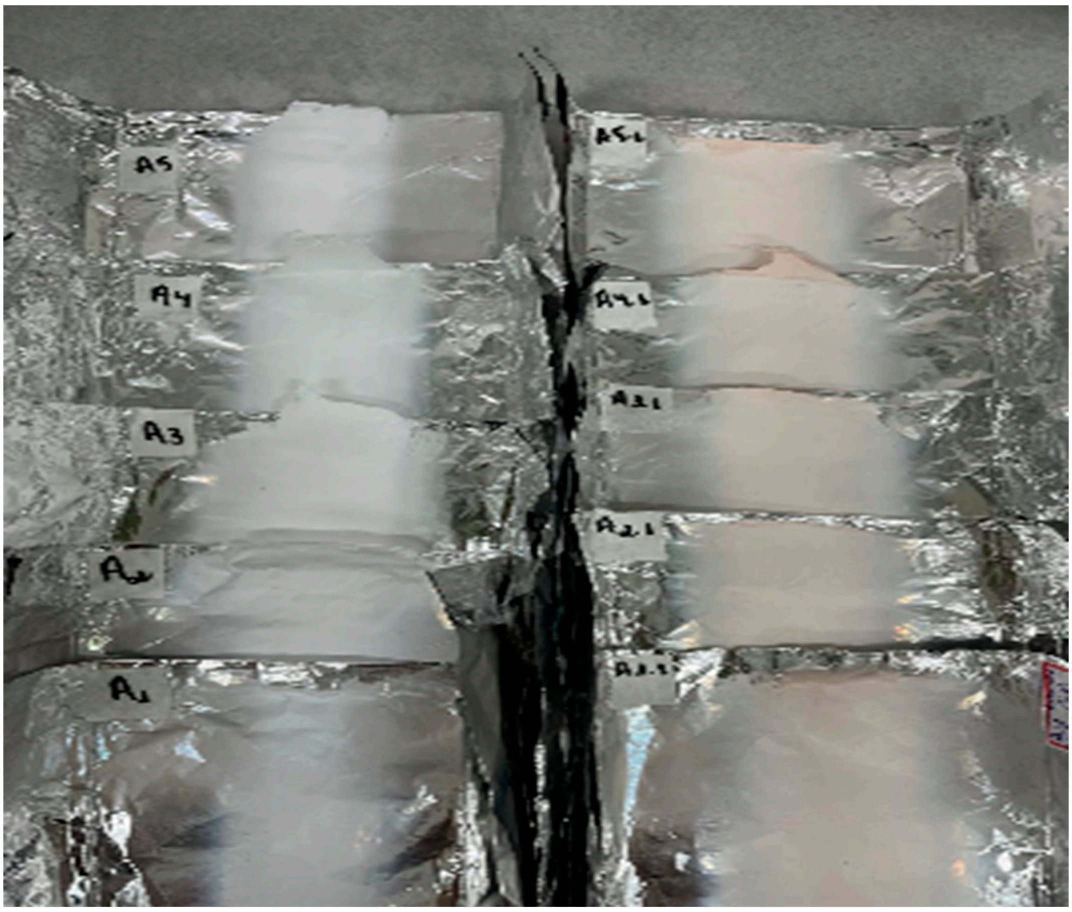

Figure S1. Representative photographs of the electrospun nanofibrous mats deposited on the aluminum support after electrospinning, illustrating their visual integrity and collectability.
