# Supplementary material for: Electrospun Polymeric Nanofibers Incorporating Brazilian Red Propolis Extract for Wound Dressing Applications
Source: Pharmaceutics. 2026 Jul 20;18(7):888. doi: 10.3390/pharmaceutics18070888 (PMC13414978; doi:10.3390/pharmaceutics18070888)
Supplement: Supplementary file 1 [file pharmaceutics-18-00888-s001.zip › pharmaceutics-4349290-Supplementary S2.pdf]

pharmaceutics

Supplementary Materials S2

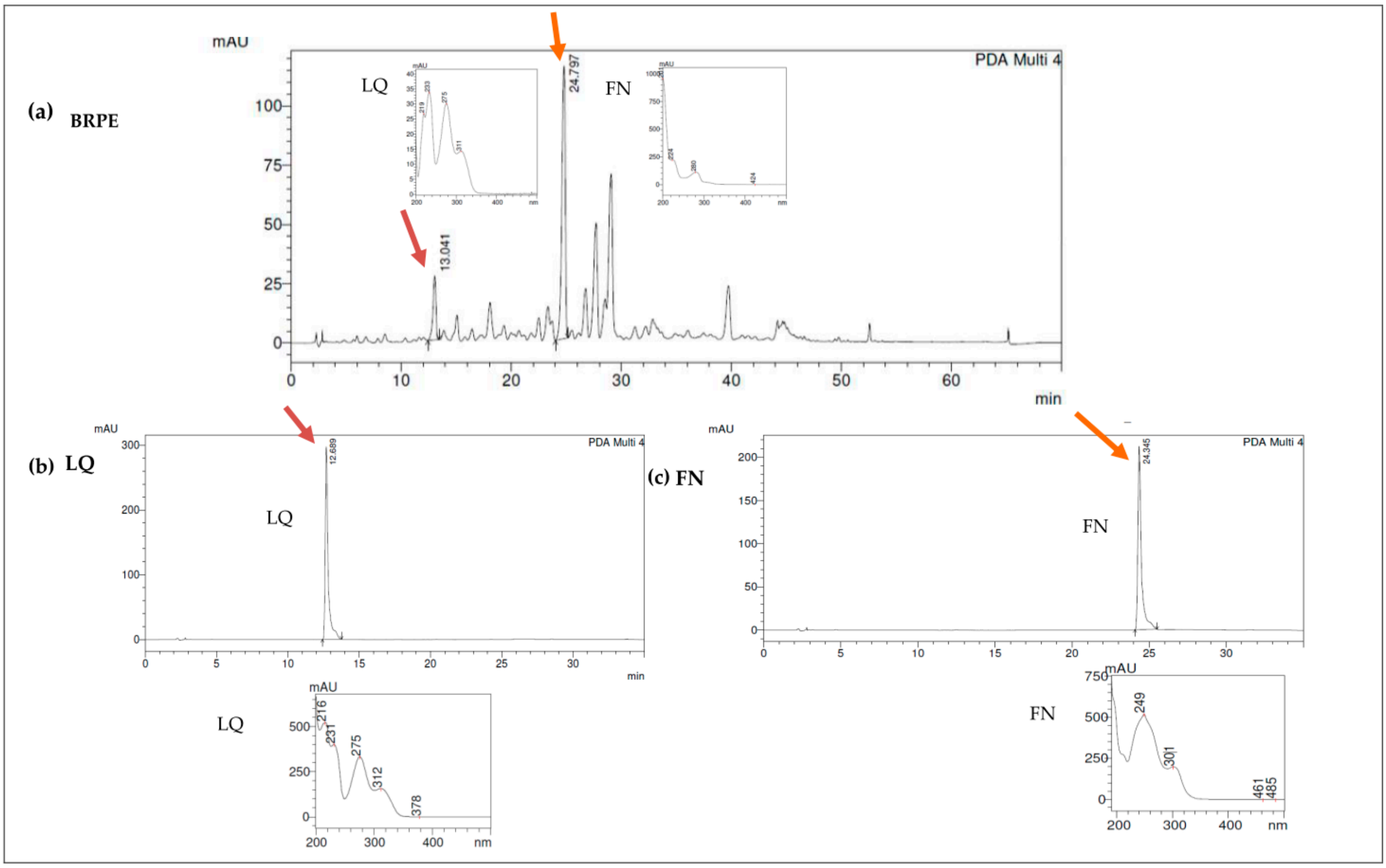

Figure S2. Representative HPLC-DAD chromatograms recorded at 281 nm and UV spectra used for qualitative confirmation of BRPE marker signals. Panels: (a) BRPE chromatogram and UV spectra of the LQ and FN-associated peaks; (b) liquiritigenin standard and UV spectrum; and (c) formononetin standard and UV spectrum.

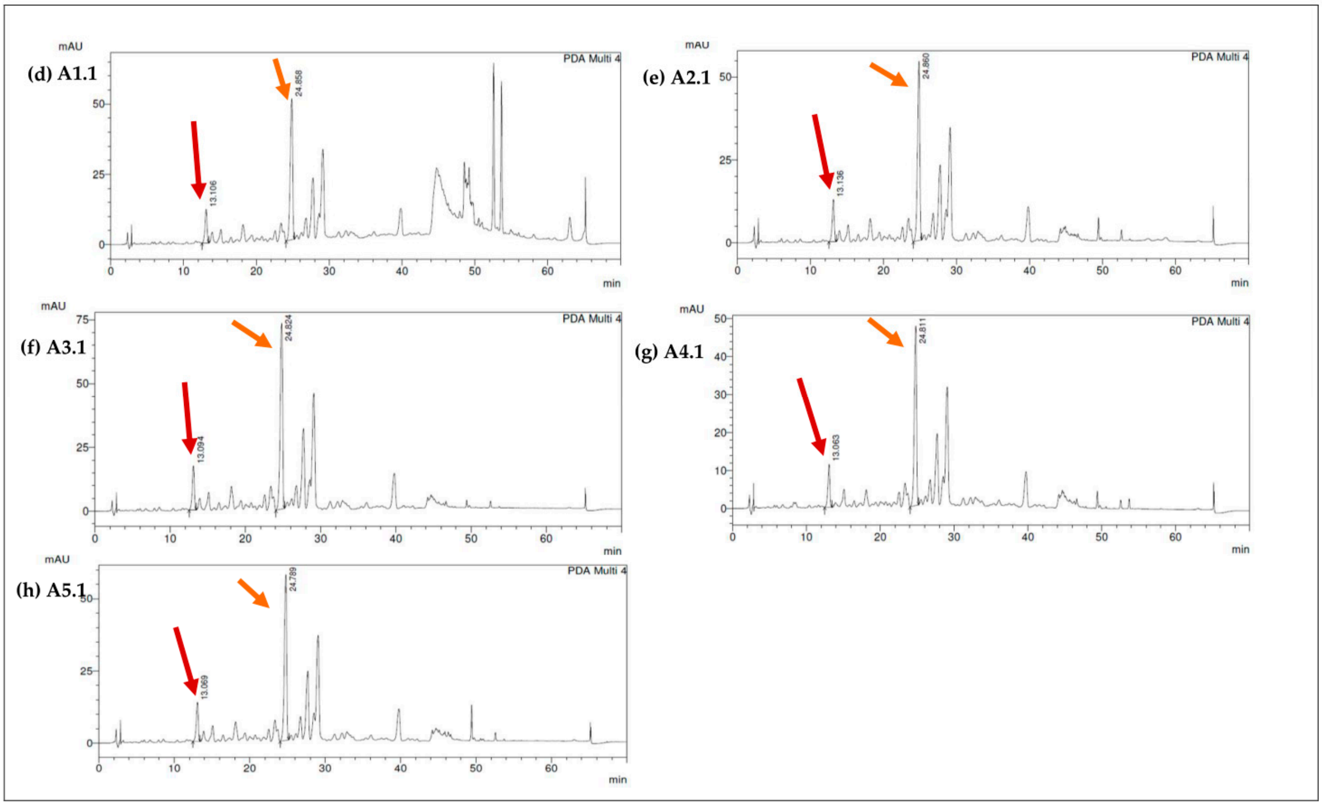

Figure S3. Figure S3. Representative HPLC-DAD chromatograms recorded at 281 nm and UV spectra used for qualitative confirmation of BRPE marker signals in the electrospun nanofibers. Panels: (d) A1.1; (e) A2.1; (f) A3.1; (g) A4.1; and (h) A5.1. The BRPE chromatogram showed two major peaks at 13.041 and 24.797 min, which were preserved in all loaded nanofibers (13.063–13.136 and 24.789–24.860 min, respectively). The analytical standards showed retention times of 12.689 min for liquiritigenin and 24.345 min for formononetin. The DAD spectra of the standards exhibited characteristic absorption maxima at approximately 216, 231, 275, 312, and 378 nm for liquiritigenin, and 249, 301, 461, and 485 nm for formononetin.

Table S2. Summary of HPLC-DAD quantification and retention efficiency of liquiritigenin (LQ) and the formononetin-associated marker signal (FN) in BRPE-loaded electrospun nanofibers.

**Sample**	**LQ (% d.b.)**	**LQ retention efficiency (%)**	**FN (% d.b.)**	**FN retention efficiency (%)**
A1.1	0.134 ± 0.010	95 ± 7	0.771 ± 0.049	92 ± 6
A2.1	0.132 ± 0.002	93 ± 2	0.761 ± 0.012	91 ± 1
A3.1	0.142 ± 0.001	101 ± 1	0.816 ± 0.006	98 ± 1
A4.1	0.113 ± 0.005	80 ± 3	0.638 ± 0.030	76 ± 4
A5.1	0.134 ± 0.003	95 ± 2	0.774 ± 0.018	93 ± 2

Values are expressed as mean ± standard deviation. Retention efficiency (%) was calculated as the ratio between experimental and theoretical marker content multiplied by 100.

Table S3. Replicate analytical values used for marker quantification and retention-efficiency calculations.

Liquiritigenin (LQ)

Sample	Replicates	Marker content (% w/w d.b.)	Retention efficiency (%)	Theoretical content (% w/w d.b.)
A1.1	1	0.13	92	0.141
A1.1	2	0.15	103	0.141
A1.1	3	0.13	90	0.141
A2.1	1	0.13	94	0.141
A2.1	2	0.13	94	0.141
A2.1	3	0.13	91	0.141
A2.1	4	0.13	94	0.141
A3.1	1	0.14	100	0.141
A3.1	2	0.14	101	0.141
A4.1	1	0.12	82	0.141
A4.1	2	0.11	75	0.141
A4.1	3	0.11	81	0.141
A4.1	4	0.12	82	0.141
A5.1	1	0.14	97	0.141
A5.1	2	0.13	93	0.141
A5.1	3	0.14	97	0.141

Formononetin (FN)

Sample	Replicate	Marker content (% d.b.)	Retention efficiency (%)	Theoretical content (% d.b.)
A1.1	1	0.75	89	0.837
A1.1	2	0.83	99	0.837
A1.1	3	0.74	88	0.837
A2.1	1	0.77	92	0.837
A2.1	2	0.76	91	0.837
A2.1	3	0.74	89	0.837
A2.1	4	0.77	91	0.837
A3.1	1	0.81	97	0.837
A3.1	2	0.82	98	0.837
A4.1	1	0.66	79	0.837
A4.1	2	0.59	71	0.837
A4.1	3	0.64	77	0.837
A4.1	4	0.66	79	0.837
A5.1	1	0.79	94	0.837
A5.1	2	0.75	90	0.837
A5.1	3	0.78	94	0.837
